# Using accelerometers for tracking loggerhead and green sea turtle behaviour

**DOI:** 10.1186/s40317-025-00415-3

**Published:** 2025-06-18

**Authors:** Jessica Harvey-Carroll, Daire Carroll, Jose Luis Crespo-Picazo, Daniel García-Párraga, David March

**Affiliations:** 1 Department of Biological and Environmental Sciences, University of Gothenburg, Gothenburg, Sweden; 2 Gothenburg Global Biodiversity Centre, Gothenburg, Sweden; 3 Conservation Department, Fundación Oceanografic de la Comunitat Valenciana, Valencia, Spain; 4 Cavanilles Institute of Biodiversity and Evolutionary Biology, University of Valencia, Valencia, Spain; 5 Centre for Ecology and Conservation, University of Exeter, Exeter, UK

## Abstract

**Background:**

Understanding animal behaviour is critical for the design of effective conservation and management strategies. Animal-borne tri-axial accelerometers constitute a type of biologging device which have the potential to provide continuous high-resolution behavioural data. For marine animals, device attachment position may influence both the accuracy of behavioural predictions and the hydrodynamic profile of the animal. We present a case study on the use of accelerometers for the behavioural classification of two sea turtle species in captivity: the loggerhead (*Caretta caretta*) and green (*Chelonia mydas*) turtle. Accelerometers were placed on the first and third scute to represent extreme placement scenarios. We trained Random Forest (RF) models to classify behaviour and assessed the impact of placement and sampling frequency on accuracy. In addition, we assessed the impact of device position on carapace drag coefficient using Computational Fluid Dynamics (CFD).

**Results:**

We achieved a high accuracy for behavioural classification (0.86 for loggerhead and 0.83 for green turtles). We determined that overall RF accuracy for both species is significantly higher for devices positioned on the third scute compared to the first scute (*P* < 0.001) and with a smoothing window of 2 s compared to 1 s (*P* < 0.001). We found no significant effect of sampling frequency and therefore recommend the use of 2 Hz in future work to optimise battery life and device memory. CFD modelling indicated an increase in drag coefficient from a maximum of 0.028 without a device to a maximum of 0.064 with a device for an isolated turtle carapace. Attachment to the first scute significantly (*P* < 0.001) increased drag coefficient relative to the third scute.

**Conclusions:**

Moving forward, the attachment and sampling protocols we present here may be adopted in future studies involving captive sea turtles. Further research is needed to assess their applicability and effectiveness under free-ranging conditions to enable their use in wild populations.

**Supplementary Information:**

The online version contains supplementary material available at 10.1186/s40317-025-00415-3.

## Introduction

Tri-axial animal-borne accelerometers (hereafter referred to as accelerometers) are an increasingly affordable and popular form of biologging device [1, 2]. Accelerometer readouts can be used to infer animal behaviour [3]. This can be achieved by the process of ground truthing, during which tri-axial acceleration data are matched to observed behaviours [2, 4]. The processing of accelerometer data can then be readily automated [5–7]. Accelerometers circumvent many of the challenges traditionally associated with studying animal behaviour in an undisturbed state (e.g. the observer effect) and allow for continuous behavioural monitoring at high temporal resolution [4, 6, 8].

Animal-borne devices can potentially impact behaviour and survival [9, 10]. This has implications for both study validity and animal welfare [11]. For marine species, the increased drag associated with device attachment is a particular consideration [12–14]. Changes in drag coefficient can be determined experimentally [12, 14, 15] or through computational fluid dynamics (CFD) [13, 16, 17]. CFD involves solving a sequence of partial differential equations expressing local balances of mass, momentum, and energy. It enables the interaction of a three-dimensional body with a flow of surrounding media to be simulated [13, 14].

Despite their widespread use, there is a lack of standardisation of accelerometer attachment protocols and device settings (e.g. sampling frequency and window length; the smallest unit of time for which behaviour is analysed) within study species [2, 4]. There is often no justification given for choice of settings [4]. Settings, along with placement and attachment type, can affect the accuracy of behavioural inference [18]. Hence, to optimise data collection and minimise the impact on the animal, species-specific standard protocols are needed [17, 19, 20].

Loggerhead (*Caretta caretta*, IUCN status = vulnerable) and green (*Chelonia mydas*, IUCN status = endangered) turtles are frequently admitted into rehabilitation centres as a result of stranding, bycatch, and/or entanglement [21, 22]. During rehabilitation, monitoring of health and behaviour is vital for ensuring successful release [21–23]. Accelerometers have the potential to streamline such monitoring [24, 25]. Devices can be easily attached to the carapace of hard-shelled sea turtles using an adhesive [26]. To date, there has been no standardisation of accelerometer positions and settings for captive sea turtles.

In this case study, we present a comparison of accelerometers positioned on the first and third vertebral scute for loggerhead and green turtles in captivity. Through ground truthing, we develop automated classifiers to infer behaviour based on accelerometer readouts. We optimise sampling frequency and window length to maximise the accuracy of behavioural classification. We then compare accuracy between positions and estimate the cost to the animal in terms of changes to drag coefficient based on CFD modelling. Moving forward, this work can be used to inform the development of standardised protocols for studying sea turtle behaviour using accelerometers.

## Methods

**Fig. 1:**
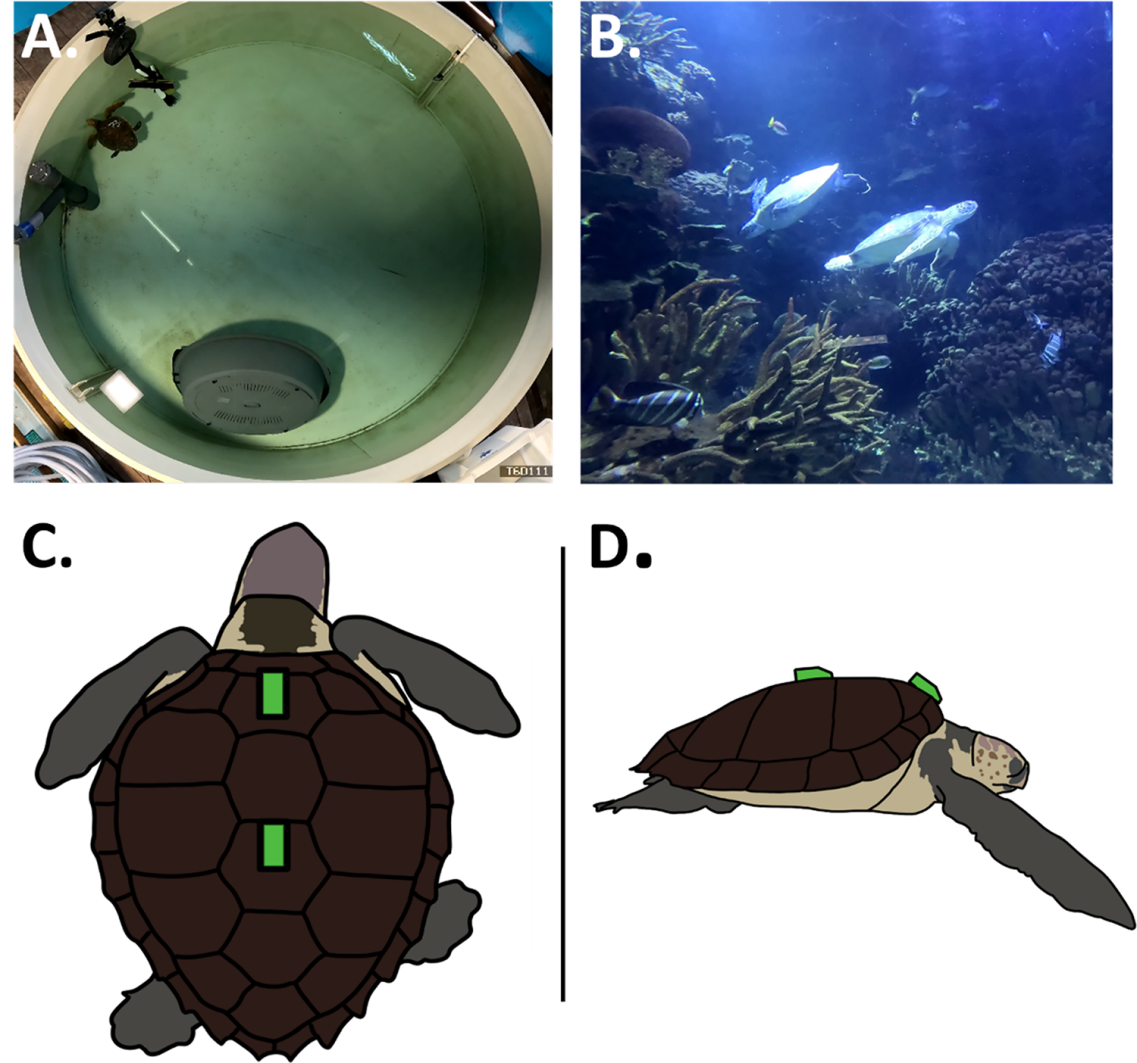
**A** Loggerhead turtle tank with enrichment devices as per [27]. **B** Section of Green turtle tank (“*Oval Room*”)*.* **C** Diagram with positions of accelerometers shown dorsally and **D** laterally

A set of experiments were conducted to assess the impact of tag attachment position on behavioural classification and drag coefficient. Experiments were conducted using seven loggerhead and eight green turtles (Supplementary information, Table S1, Fig. 1A, B).

### Behavioural data collection

#### Accelerometer attachment and configuration

Two accelerometers (Axy-trek Marine, TechnoSmart Europe, 21.6 g) were attached to each turtle’s carapace. Accelerometers were attached proximally to the first and third scutes, to represent extreme differences in attachment within the literature (Fig. 1C, D). Attachment sites were first cleaned with 70% ethanol and dried. VELCRO® was then superglued to both the scute and the accelerometer. The accelerometer was then sealed to the shell using T-Rex® waterproof tape.

During a pilot deployment, acceleration values did not exceed 4 g and 2 g for loggerhead and green turtles, respectively. During subsequent deployments, accelerometers were configured to record 100 Hz data at 8-bit resolution with a dynamic range of ± 2 g for loggerhead and ± 4 g for green turtles (Table S2).

#### Behavioural recording and ethogram creation

Turtle behaviour was recorded with both accelerometers attached simultaneously. For loggerhead turtles, a GoPro Hero 11 was fixed above the tank to allow for continuous recording [27]. Green turtle behaviour was recorded using one of two systems: (I) by following individuals with a GoPro Hero 11 mounted on a telescopic pole, and (II) by Little Leonardo DVL400M130 animal-borne video cameras attached to the carapace (Table S1; Figure S1). A total of 66,783.82 s of video footage was obtained (45,804.2 s for loggerhead and 20,979.62 s for green turtles).

For synchronisation of annotated behaviours and accelerometer readouts, videos were synchronised to UTC by recording time on either https://time.is/, or a GPS app (GPS test, Chartcross Limited) [2, 28]. Behavioural ethograms (Table S3) were generated by a single observer based on recorded videos using BORIS [2, 29]. 18 and 14 initial behaviours were defined for loggerhead and green turtles, respectively, based on previous published ethograms and observation of videos (Table S3) [30]. For videos obtained using animal-borne video cameras, only behaviours involving the head were labelled (“*biting*”, “*breathing*”, *and* “*surface biting*”) (Table S3). Accelerometer readouts were then labelled with behaviours. A total of 46,723.87 s of behaviour was labelled (32,786.75 and 13,937.12 s for loggerhead and green turtles, respectively). The first and last second of each behaviour was omitted to account for time synchronisation errors [2, 28]. 

### Behavioural data analysis

#### Behavioural classification

Segmenting accelerometer data into different window lengths is a commonly used method for calculation of summary metrics [4]. To investigate the effect of window length on behavioural inference, each segment of behaviour was split into equal blocks of 1 and 2 s in length. If a behaviour did not last 2 s, only 1 s durations were used (e.g. “*breathing*” for loggerhead turtles). For each window length, the data were resampled from the original 100 Hz to produce 50, 25, 12, 10, 8, 4, and 2 Hz datasets [3, 28]. Accelerometer summary metrics (n = 18, Table S4) were calculated for each dataset [2, 31, 32].

To automatically classify behaviour, a random forest (RF) model was used. RFs are supervised machine learning algorithms which use decision trees [33]. Datasets were randomly split into training (70%) and testing (30%) sets using the *createDataPartion()* function, ensuring balanced distributions of behaviour classes between the two datasets. Data from the same individual were included in both the training and testing set. If a behaviour occurred in three or less individuals, it was excluded from the RF model to allow for a robust cross-validation design (Table S3) [34, 35]. Ten and six behaviours were used for 1-s windows, and eight and five behaviours for 2-s windows, for loggerhead and green turtles, respectively. Separate RF models were trained on datasets for each species, window length, and sampling frequency.

During model tuning and performance evaluation individual based k-fold cross-validation (sevenfold and eightfold for loggerhead and green turtles, respectively) and up-sampling (random resampling with replacement for minority behaviours with the smallest number of windows, ensuring an even distribution of behavioural segments) were conducted for the training dataset [2]. In order to account for individuals (repeated measures structure) a block factor in the leave-one-out cross-validation process was included. Individuals were used as folds. This ensured all data from a single individual were iteratively excluded from the training dataset and used for validation [2, 35–38].

Models were iteratively run with varying number of predictors, and a maximum of 1000 trees. The area under the receiver operating curve (AUC) was calculated for each model to evaluate performance, with high AUC indicating a better performing model. The parameterised RF models were then applied to testing datasets and overall accuracy was calculated using the *confusionMatrix()* function in the R-package *caret* [2]. For final RF models, the relative importance of summary metrics to accuracy and predictive ability were determined based on the impurity variable importance mode [39]. RF fitting and evaluation was carried out using the R-packages *caret* [40] and *ranger* [39].

#### Effect of tag position on classification

To assess the effect of tag position on overall RF model accuracy, a beta regression was conducted. Frequency (“*Frequency*”), device position (“*Position*”) and species (“*Species*”) were included as main effects with an interaction. Window length (“*Window*”) was included as a main effect without interaction. Overall RF accuracy (“*Accuracy*”) was run as the dependant variable. Where significance was found, pairwise post hoc testing was conducted using the R-package *emmeans* [41, 42]. To assess the effect of tag position on RF model balanced accuracy (an average measure of correct recall for individual classes [43, 44]) for classification of individual behaviours, separate beta regressions were conducted for each species. As only position-specific differences within behaviours were of interest, each combination of behaviour and scute position was treated as a unique level for the independent variable (“*Behaviour.position*”). For both models, balanced accuracy (“*Balanced Accuracy*”) was included as the dependent variable. A fixed window length of 2 s was selected. Where significance was found, pairwise post hoc testing was conducted using the R-package *emmeans* [41, 42].

To assess the effect of sampling frequency (“*Frequency*”) on RF model balanced accuracy of individual behaviours, a beta regression model was conducted for each species. For each model, balanced accuracy (“*Balanced Accuracy*”) was included as the dependent variable. Sampling frequency (“*Frequency*”) and behaviour (“*Behaviour*”), were used as main effects with interactions. A fixed window length of 2 s was selected.

### Effect of tag position on drag coefficient

Drag coefficient (*C*_*d*_) is a positive dimensionless constant that quantifies the resistance of an object to a surrounding fluid. The higher the drag coefficient, the more drag an object will experience when moving [13]. It can be calculated based on drag force (*D*, N), fluid density (*ρ* kg m^−3^), velocity (*U* m s^−1^), and frontal area (*A*, m^2^) based on:

$$C_{d} = \frac{D}{{0.5\rho U^{2} A}}.$$

#### Three-dimensional digital mesh construction

To construct a three-dimensional digital model (a mesh) of a turtle carapace for use in computational fluid dynamics (CFD), images of an individual loggerhead turtle (individual OG734, Table S1) with two attached devices were collected outside the tank (Figure S2). Using a GoPro Hero 11, 427 images of the turtle’s carapace were taken from approximately 30 cm away with different angles covering the entire carapace. The curved length (0.43 m), straight length (0.39 m), curved maximum width (0.41 m), and straight maximum width (0.34 m) of the carapace were recorded. Images were imported into WebODM 1.9.15 and a three-dimensional mesh was created using the following settings:

Auto-boundary: true, mesh-octree-depth: 12, use-3dmesh: true, pc-quality: high, mesh-size: 300,000.

As the turtle was moving its head and limbs, the mesh poorly represented these areas, however the carapace was well represented. The mesh was imported into Blender 3.3.0. Three new meshes representing the area of carapace dorsal to the marginal scutes: (I) with no device, (II) with a single device in the first scute position, and (III) with a single device in the third scute position were created (Figure S2). Meshes were scaled so that their curved length, straight length, curved diameter, and straight diameter matched that of the original turtle’s carapace (Table S1).

#### Computational fluid dynamics

CFD was used to estimate the drag coefficient of a turtle carapace with and without attached devices by simulating the movement of the animal through sea water. CFD was carried out in ANSYS Fluent R2 using a k-ξ model [13, 14, 16]. A symmetrical fluid domain 2 m in length, 1 m in width and 1 m in height was established. Carapace geometry was subtracted from the base of this fluid domain. The walls of the fluid domain cranially and caudally to the carapace were set to represent velocity inlets and pressure outlets, respectively. All other walls, including those representing the carapace, were set as solids. A mesh of approximately 1 × 10^6^ cells, the majority of which were located close to the carapace, was generated. The mesh was refined down to an average *y* + value of 1 to resolve the flow at the viscous sublayer [13, 16]. Fluid density and viscosity were set to represent sea water (1.06 e−6 m^2^ s^−1^ and 1.028 kg m^−3^, respectively) [13].

Drag is expected to decrease with pressure (force per unit surface area) but increase with velocity (rate of movement) [13, 45]. Simulations were run at a pressure of 145,000 kPa, representing a depth of five m (85% of the dives reported for juvenile loggerhead turtles [46]). For each of the three three-dimensional meshes (no device, 1st scute, and 3rd scute) simulations were run with flow velocities of 0.2, 0.4, 0.6, 0.8, 1, and 1.2 m s^−1^, representing a realistic range of sea turtle velocity [47]. A drag coefficient was determined for each carapace from each simulation.

To test for a significant effect of tag position on drag coefficient, a linear regression was conducted taking drag coefficient (“*Cd*”) as the dependent variable, with device position (“*Position*”) and velocity (“*Velocity*”) as independent variables with an interaction between position and velocity. Pairwise post hoc testing was then conducted using the *emmeans* package in R.

#### Data handling and availability

Unless otherwise specified, data processing and visualisation was conducted in R version 4.3.2 [48] with data organisation assisted by the *data.table, lubridate, zoo,* and *tidyverse* packages. Data and code will be available upon publication on GitHub: https://github.com/JessHCarroll/Turtle_ACC_analysis and archived on Zenodo 10.5281/zenodo.14003216.

## Results

### Optimal sampling frequency and window length

**Fig. 2:**
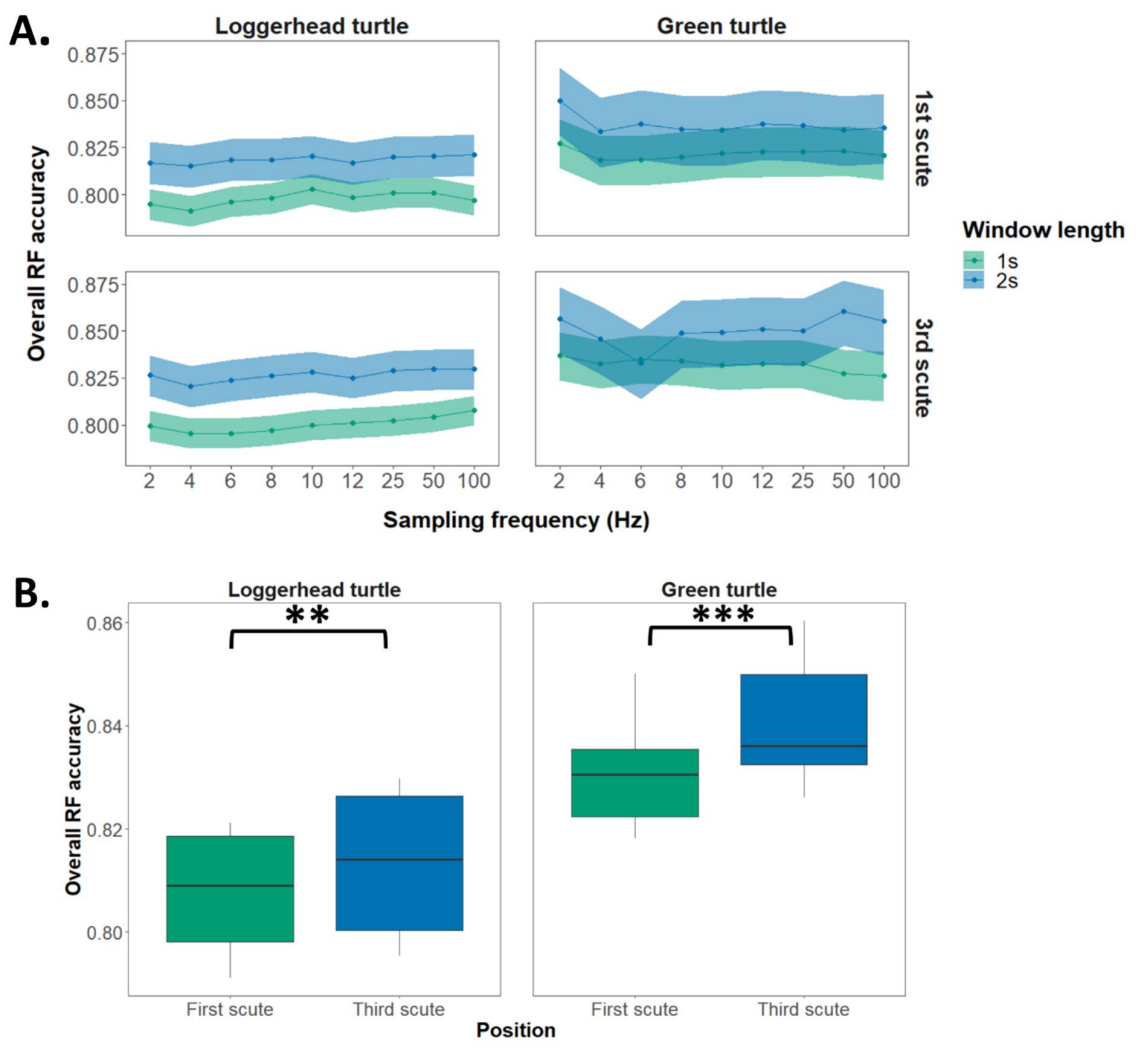
**A** Overall RF accuracy for classification of behaviour across frequencies between 1 (blue) and 2 (green) s window lengths. Shaded regions represent 95% confidence intervals. **B** Comparison of accuracy of RF behavioural classification between first (green) and third (blue) scutes. Loggerhead turtles *n* = 7, green turtles *n* = 8. Boxplots depict median relative accuracy levels and the 25th and 75th percentiles. Whiskers are 1.5× the interquartile range. *** indicates a *P*-value < 0.001, ** indicates a *P*-value < 0.01

No significant affect of sampling frequency on overall RF model accuracy for classification of behaviours was found (Estimate = − 4 e−05, SE = 2 e−04, *Z*-value = − 0.251, *P* = 0.802). Frequencies of both 100 and 2 Hz resulted in overall RF accuracies of 0.83 for loggerhead and 0.86 for green turtles with a 2-s window length (Fig. 2A). No interaction between species and frequency was identified (Estimate = 4 e−04, SE = 2 e−04, *Z*-value = 1.823, *P* < 0.068).

A significant interaction between position and species was identified (Estimate = − 0.050, SE = 0.014, *Z*-value = − 3.597, *P* < 0.001). Post hoc analysis revealed that attaching the accelerometer to the third scute resulted in significantly higher levels of overall accuracy for both loggerhead (first vs third scutes: Estimate = − 0.005, SE = 0.001, DF = ∞, *Z*-ratio = − 3.533, *P* = 0.002) and green turtles (first vs third scute*:* Estimate = − 0.012, SE = 0.001, DF = ∞, *Z*-ratio = − 8.300, *P* < 0.001) (Fig. 2B).

Accelerometer position (Estimate = 0.085, SE = 0.010, *Z*-value = 8.298, *P* < 0.001) and species (Estimate = − 0.154, SE = 0.011, *Z*-value = − 13.636, *P* < 0.001) were both found to be significant predictors of overall accuracy.

For RF models trained on datasets with window lengths of 2 s and sampling frequencies of 2 Hz, Vectorial Dynamic Body Acceleration (VeDBA), a metric used to estimate energy expenditure and activity levels, was a crucial predictor for both species, with higher importance for green turtles. Additionally, movement variability, such as the standard deviation of roll (SD roll), was particularly important for loggerhead turtles (Figure S3).

### Individual behaviours

**Fig. 3:**
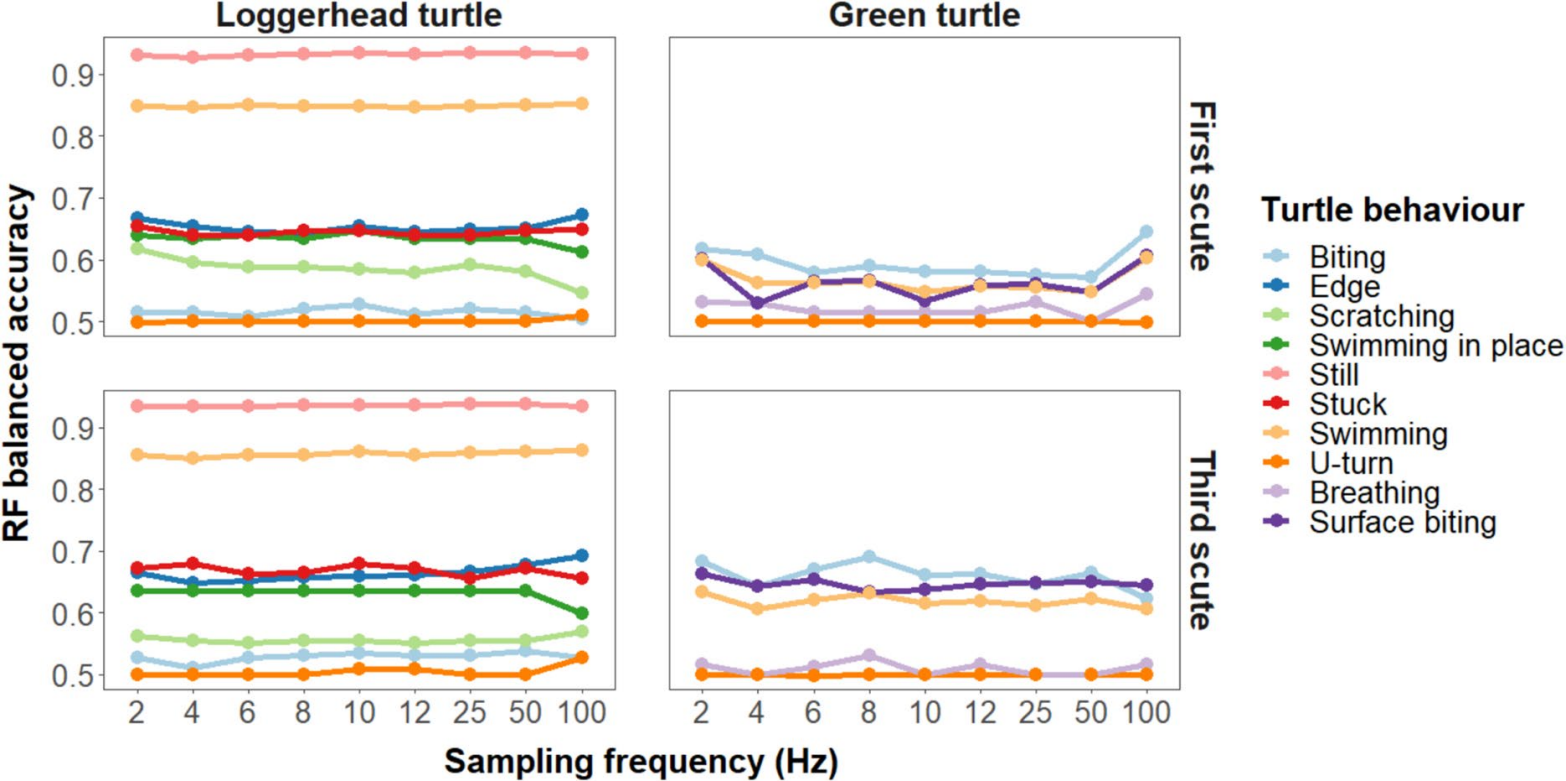
Balanced accuracy for discrete behaviours used in RF classification models for a window length of 2 s

For individual loggerhead turtle behaviours, sampling frequency was found to significantly affect the balanced accuracy of *“edge”* and *“swimming in place”* only (“*edge*”: Estimate = 0.001, SE = 5 e−04, *Z*-value = 2.969, *P* = 0.003. “*swimming in place*”: estimate = − 0.001, SE = 5 e−04, *Z*-value = − 2.408, *P* = 0.016. Tables S5, Fig. 3).

Accelerometer location significantly affected the balanced accuracy of *“scratching”* (Estimate = 0.030, SE = 0.004, DF = ∞, *Z*-ratio = 7.345, *P* < 0.001) and “*stuck*” behaviours (Estimate = − 0.023, SE = 0.004, DF = ∞, *Z*-ratio = − 5.945, *P* < 0.001. Tables S6 & S7, Figure S6A).

For green turtle behaviours, sampling frequency was not found to significantly affect the balanced accuracy of any behaviours (Tables S8, Fig. 3). Accelerometer position was found to significantly affect the balanced accuracy of *“biting”, “surface biting”* and “*swimming”* behaviours (*“Biting”:* Estimate = − 0.066, SE = 0.007, DF = ∞, *Z*-ratio = − 9.247, *P* < 0.001. “*Surface biting*”: Estimate = − 0.083, SE = 0.007, DF = ∞, *Z*-ratio = − 11.573, *P* < 0.001. “*Swimming*”: Estimate = − 0.052, SE = 0.007, DF = ∞, *Z*-ratio = − 7.171, *P* < 0.001. Tables S9 & S10, Figure S6B).

### Effect of tag position on drag

**Fig. 4:**
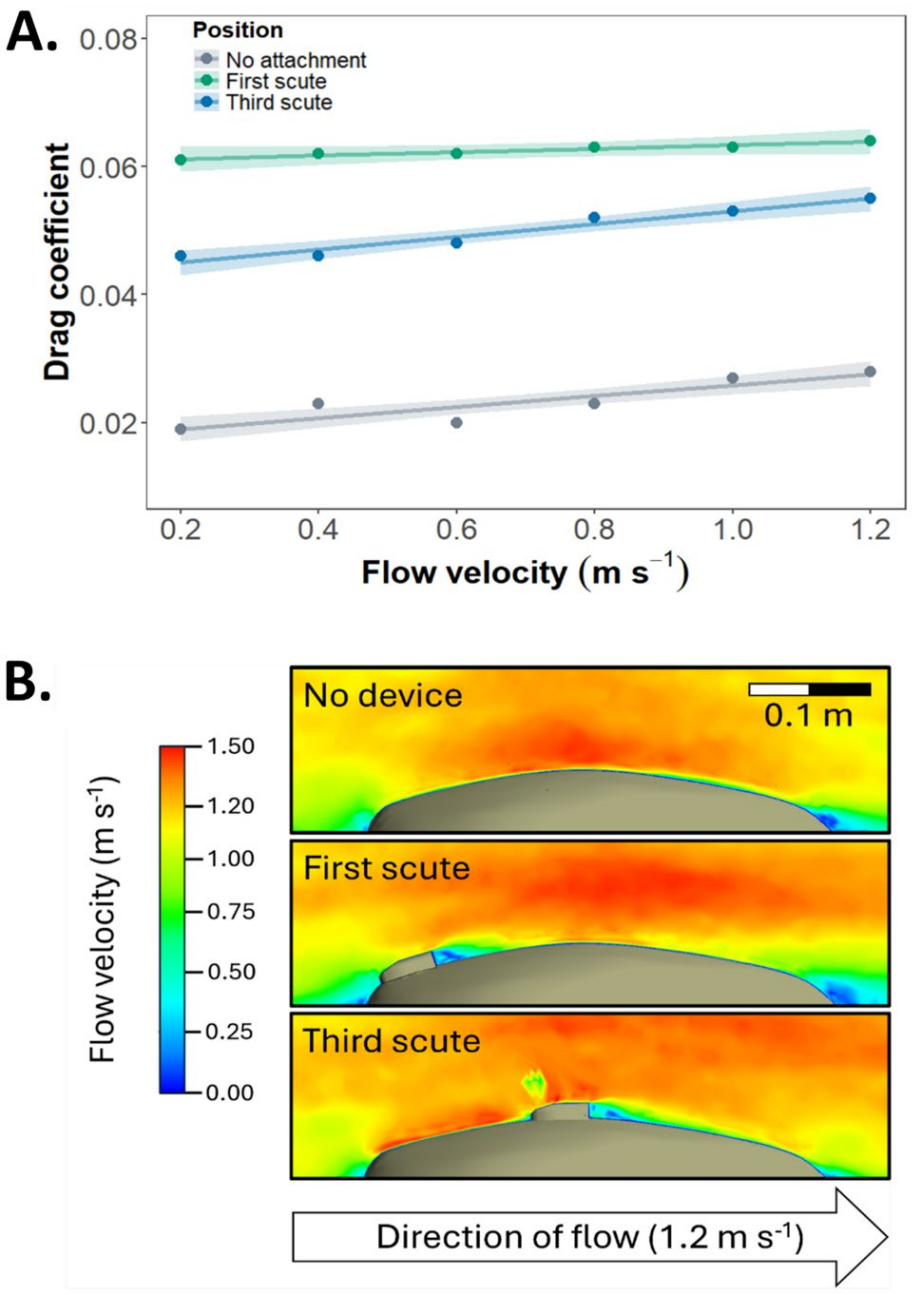
**A** Drag coefficients for turtle carapaces without a device (grey) and with a device on the first (green) and third (blue) scutes based on CDF modelling. Lines and shaded regions represent predictions, and 95% confidence intervals based on linear regression. **B** Visualisation of flow velocity for a turtle carapace (grey region) in a 1.2 m s^−1^ flow based on CDF modelling

CFD modelling for a carapace without an attached device predicted a relatively constant drag coefficient of between 0.019 and 0.028 at all velocities (Fig. 4A). Carapaces with devices had higher drag coefficients (between 0.046 and 0.064, a difference of between 96 and 221%) at all velocities (Fig. 4A). Velocity was found to be a significant predictor of drag coefficient for the carapace without a device (slope = 0.009, SE = 0.002, *t*-value = 5.816, *P* < 0.001) and for the carapace with a device on the first scute (slope = 0.003, SE = 0.002, *t*-value = − 2.81, *P* = 0.016) but not the third scute (slope = 0.01, SE = 0.002, *t*-value = 0.685, *P* = 0.506).

Post hoc analysis indicated a significant difference in drag coefficient between all three carapaces, with the highest estimated drag coefficient for the first scute, followed by the third scute, and finally no attachment (Table 1; Fig. 4B).

**Table 1:** Pairwise comparison for position of accelerometer on drag coefficient

Contrast	Estimate	SE	DF	*t*-ratio	*P* value
No attachment v. first scute	− 0.039	7 e−4	12	− 55.013	< 0.001
No attachment v. third scute	− 0.027	7 e−4	12	− 37.456	< 0.001
First scute v. third scute	0.013	7 e−4	12	17.557	< 0.001

## Discussion

We have shown that the positioning of accelerometers on captive loggerhead and green turtles can significantly impact the accuracy of behavioural classification from accelerometer readouts. This should be a consideration in the design of future studies. Attaching accelerometers proximally to the turtle’s third scute resulted in significantly more accurate overall behavioural classification. When the balanced accuracy of individual behaviours was assessed, “*biting*”, “*surface biting*” and “*swimming”* behaviours (green turtles) and “*scratching”* and “*stuck*” behaviours in loggerhead turtles specifically was significantly improved by positioning on the third scute. Although no significant effect of position on “*biting*” behaviour for loggerhead turtles was identified, a large increase of accuracy was seen from positioning the accelerometer on the third scute compared to the first. This may be due to the limited sample size of “*biting*” behaviours at a 2-s smoothing window. Future deployments of accelerometers on captive loggerhead and green turtles may wish to adopt the third scute as an attachment location to maximise the quality of data collected.

Our estimates of overall RF accuracy for classifying behaviours (0.83 for seven loggerhead behaviours and 0.86 for five green turtles) are in line with previous studies with smaller sample sizes [30, 49]. For example, Nishizawa et al. [49] achieved an accuracy of 86.21% for two green turtles and Jeantet et al. [30] achieved an accuracy of 79.49% for one loggerhead turtle and 86.96% for one green turtle. We chose to randomly partition data into training and testing sets based on behaviour classes due to the comparative approach taken throughout this paper. For applications of the model to new individuals, splitting data to ensure the final testing is done on new individuals may be preferable.

Larger window lengths are generally accepted to increase the accuracy of detecting complex behaviours. This comes at a cost of capturing behavioural transitions, which may result in misclassification [20, 50]. We found that window lengths of 2 s resulted in significantly higher accuracy than 1 s. Similar window lengths have been used in previous studies [30, 51, 52]. If short duration behaviours (e.g. “*biting*” or “*breathing*”) are not of interest, window length could be increased, potentially increasing RF accuracy for longer duration behaviours (e.g. “*swimming*” or “*resting*”) [20, 30, 50].

Higher classification accuracy is generally obtained with higher sampling frequencies [2, 3, 20, 53, 54]. Higher sampling frequencies, however, consume more memory space and shorten battery life [1, 3]. We found no significant interaction between overall RF accuracy and sampling frequency. This is likely due to the relatively slow movements of sea turtles compared to other marine animals [47]. Sampling frequency significantly altered the balanced accuracy for the specific behaviours “*swimming in place*” and “*edge*” in loggerhead turtles only. No other behaviours were affected. Employing a sampling frequency of 2 Hz may be preferable in future work for captive turtles as it will maximise battery life and the use of device memory.

A large range of behaviours were identified across individuals. Some of the identified behaviours, such as ‘*still*’ have ecological relevance for free-ranging turtles, who are known to rest on the seabed [55]. The ‘*Still*’ behaviour was identified with high accuracy in loggerhead turtles, regardless of positioning. Such behaviour is fundamental for initial construction of activity budgets, and in turn energetic studies [56–59]. Additionally the accompaniment of accelerometers with common depth loggers may provide important insights into specific behaviours such as resting [60–62]. Nonetheless, the full behavioural repertoire of turtles was not captured. Sea turtles in the wild will perform other behaviours of interest such as mating, responding to predators, and different foraging strategies [63–65]. Additionally, some behaviours identified in this study, such as “*edge*” are only relevant in the captive environment. Furthermore, the speed of behaviours performed may be different in captivity compared to the wild. The size of the enclosures used for loggerhead turtles was not sufficient to allow for documentation of gliding behaviour, similar to other studies using captive sea turtles [30, 66]. Despite these limitations, accelerometers have the potential to increase our understanding behaviour in captivity and more behaviours can be added in future work.

A previous study attached a variety of biologging devices to physical models of green, hawksbill, and leatherback turtles and quantified the impact on drag coefficient during wind tunnel experiments [15]. They reported drag coefficients of between 0.11 and 0.22 with increases of more than 100% for juveniles as a result of device attachment. The values of drag coefficient we report (0.02 to 0.064) for a turtle carapace based on CFD are lower than those reported for an entire turtle [15]. The maximum increase we report as a result of device attachment (221%) is also considerably higher, although our lower estimate (96%) is of a similar magnitude. The difference in overall drag coefficient is likely due to the fact that we included only the upper portion of the carapace in simulations. The use of a single simplified model excluding limbs or other parts of the body is common in CFD analysis [14, 16]. We isolated the carapace as a region of interest due to challenges in the construction of the 3D mesh from images of an unrestrained animal. Turtle head and limb orientation are likely to influences drag, influencing our results [16]. It is likely that drag coefficient for an entire turtle would be significantly higher and the overall impact of the device would be lower if the entire animal was included. Nonetheless, CFD of an isolated carapace provided a useful comparative tool for assessing the relative impact of device position on drag coefficient.

We found that drag coefficient for a carapace with a device attached in the third scute position was significantly lower than when the device was attached in the first scute position. As drag force increases proportionally with drag coefficient, such an increase is likely to impose a biologically relevant cost to the animal in terms of energetic expenditure, as supported by previous work [13, 15]. Further work is required to determine how this cost might influence animal behaviour or survival.

The device used in this study was designed to have a hydrodynamic profile (personal communications, TechnoSmart Europe). Observed differences in drag coefficient would likely increase with device size relative to carapace size and for less hydrodynamically shaped devices [14]. Individual carapace shape may also have an influence. Future work could expand on CFD modelling to include more species, more individuals of different sizes, and full turtle models in different orientations [16]. This may be aided by experimental examination of three-dimensional models in flow chambers [12, 15, 16]. In addition, to fully understand the impact of animal-borne devices more studies are needed to directly and comprehensively measure the wide-ranging effects of such devices over immediate and long term periods on free-living animals [9, 67, 68].

## Conclusion

Animal-borne tri-axial accelerometers can be used to record sea turtle behaviour with a high degree of accuracy. We have presented the first comparative study of device attachment and settings protocols for sea turtles. In future work focused on captive loggerhead and green turtles, we recommend devices to be placed on the middle line of the third scute and the use of a sampling frequency of 2 Hz. Using these settings and the RF classifiers presented, standardised recording of sea turtle behaviour in captivity can be achieved. The data from such studies hold the promise of improving our understanding of turtle behaviour, enabling rehabilitation and conservation efforts to be refined. Such information is important from both an animal welfare perspective and for future interpretation of behavioural data.

## Supplementary Information

Supplementary Material 1.

## Data Availability

Data and code is available on GitHub: https://github.com/JessHCarroll/Turtle_ACC_analysis and archived on Zenodo 10.5281/zenodo.14003216.
