# Supplementary material for: Using accelerometers for tracking loggerhead and green sea turtle behaviour
Source: Anim Biotelemetry. 2025 Jun 18;13(1):20. doi: 10.1186/s40317-025-00415-3 (PMC12177017; doi:10.1186/s40317-025-00415-3)
Supplement: Supplementary file 1 — Supplementary Material 1. [file 40317_2025_415_MOESM1_ESM.docx]

# Case report: Using accelerometers for tracking loggerhead and green sea turtle behaviour.

Jessica Harvey-Carroll^1,2^, Daire Carroll^1,2^, Jose Luis Crespo-Picazo^3^, Daniel García-Párraga^3^, and David March^4,5^.

^1^ Department of Biological and Environmental Sciences, University of Gothenburg, Gothenburg, Sweden.

^2^ Gothenburg Global Biodiversity Centre, Gothenburg, Sweden.

^3^ Conservation Department, Fundación Oceanografic de la Comunitat Valenciana, Valencia, Spain.

^4^ Cavanilles Institute of Biodiversity and Evolutionary Biology, University of Valencia, Valencia, Spain.

^5^ Centre for Ecology and Conservation, University of Exeter, United Kingdom

## Supplementary materials

Supplementary Information.

Supplementary Figures S1-S4.

Supplementary Tables S1-S10.

Supplementary References.

**Animal Information**

Loggerhead turtles (n = 7) were in the final stages of rehabilitation at Arca del Mar, Valencia, Spain. They were declared clinically healthy by a veterinarian prior to inclusion in the study. They were housed individually in cylindrical tanks of 3 m diameter and 1 m depth and kept at a temperature of 24^o^C (Figure 1A). Tanks contained enrichment devices (a brush, a shelter, and a PVC pipe) as described in [1]. Loggerhead turtles were fed a diet of herring, hake, cod, and squid.

Green turtles (n = 8) were housed in three display enclosures at the Oceanogràfic Aquarium, Valencia, Spain (Figure 1B). Two individuals (Amadeus and Gijon) were housed in the “*Oceans aquarium*” (6e6 L in volume and 5.8 m in depth, 23^o^C), four (Bubuja, Nagoya, Wasabi, and Kawasaki) were housed in “*The tunnel*” (7e5 L in volume and 3 m depth, 22 ^o^C), and two (Tack and Enoshima) were housed in the “*Oval Room*” (4.9 e 5 L in volume and 3.6 m depth, 24.5 ^o^C). Enclosures contained a range of enrichment devices (corals, rock, and marine plants) and housed several fish species. Green turtles were fed five days per week with a combination of algae gelatine (e.g. nori) and vegetables (e.g. lettuce), varying according to their stage of development.


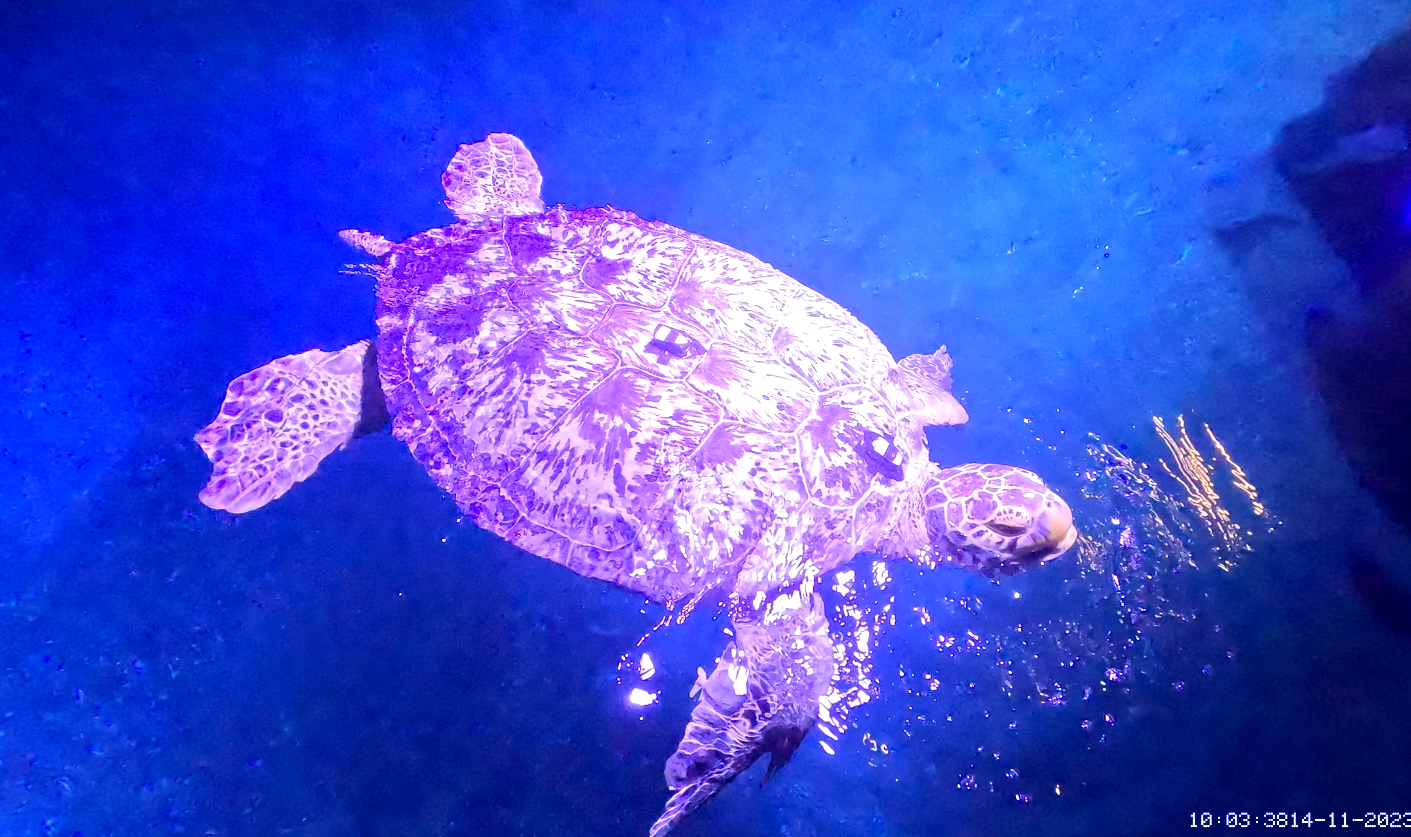

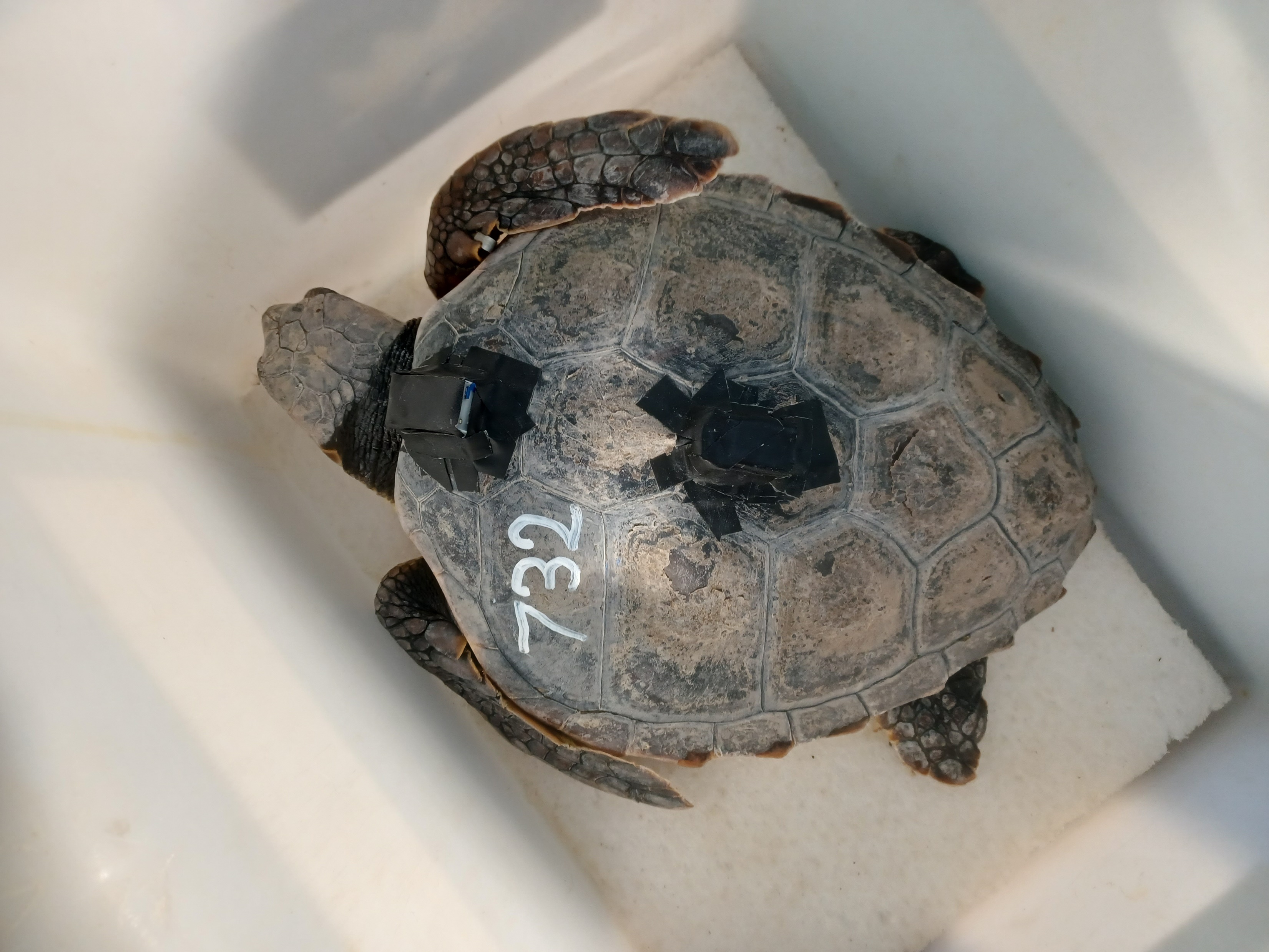


**A.**

**B.**


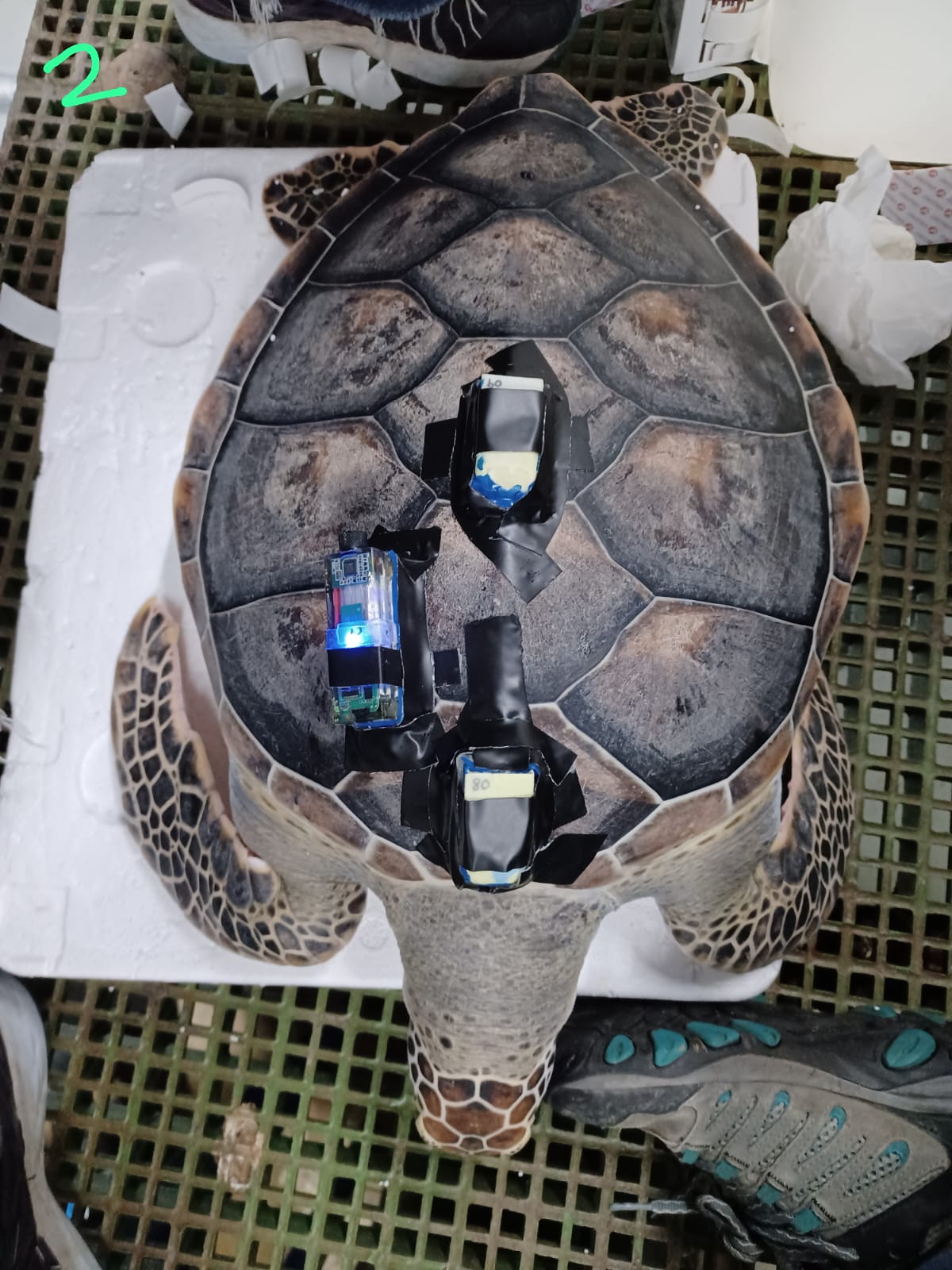


**C.**

**Figure S1**. Attachment of accelerometers to **A.** loggerhead turtle and **B.** green turtle. **C.** Green turtle with two accelerometers and an animal-borne video camera (DVL400M130, 52 g, Little Leonardo). The combined mass of the two accelerometers and animal-borne video camera constituted no more than 3 % of any individual turtle’s mass.

| 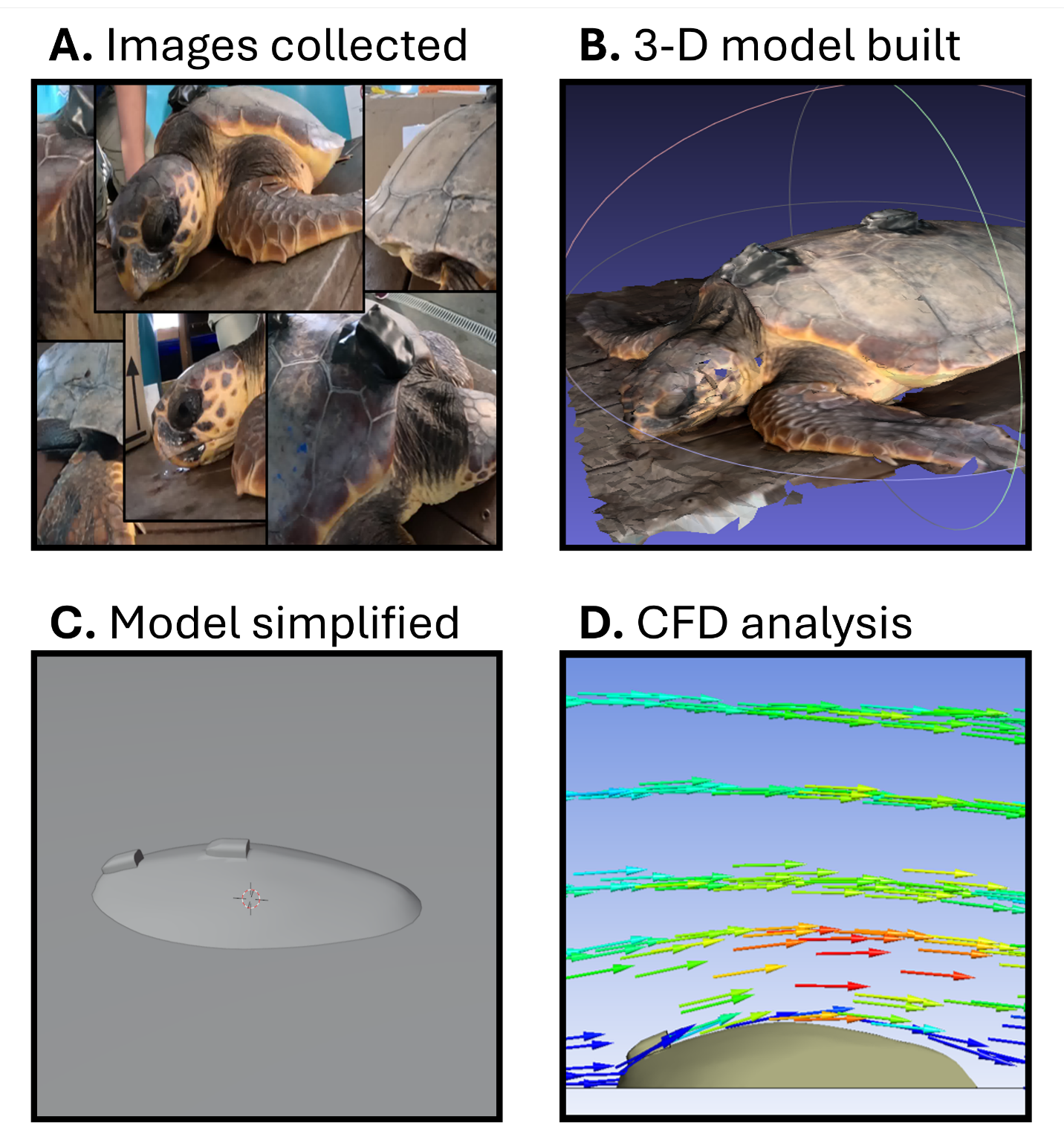 |
| --- |
| **Figure S2.** Workflow for three-dimensional and computational fluid dynamics modelling. **A.** Images of a loggerhead turtle with devices attached to the first and third scute were collected. **B.** A three-dimensional mesh of the turtle was generated. **C.** Meshes were simplified to create three-dimensional models of a turtle carapace with and without attached devices. **D.** Computational Fluid Dynamics (CFD) was used to determined drag coefficients for carapaces. |


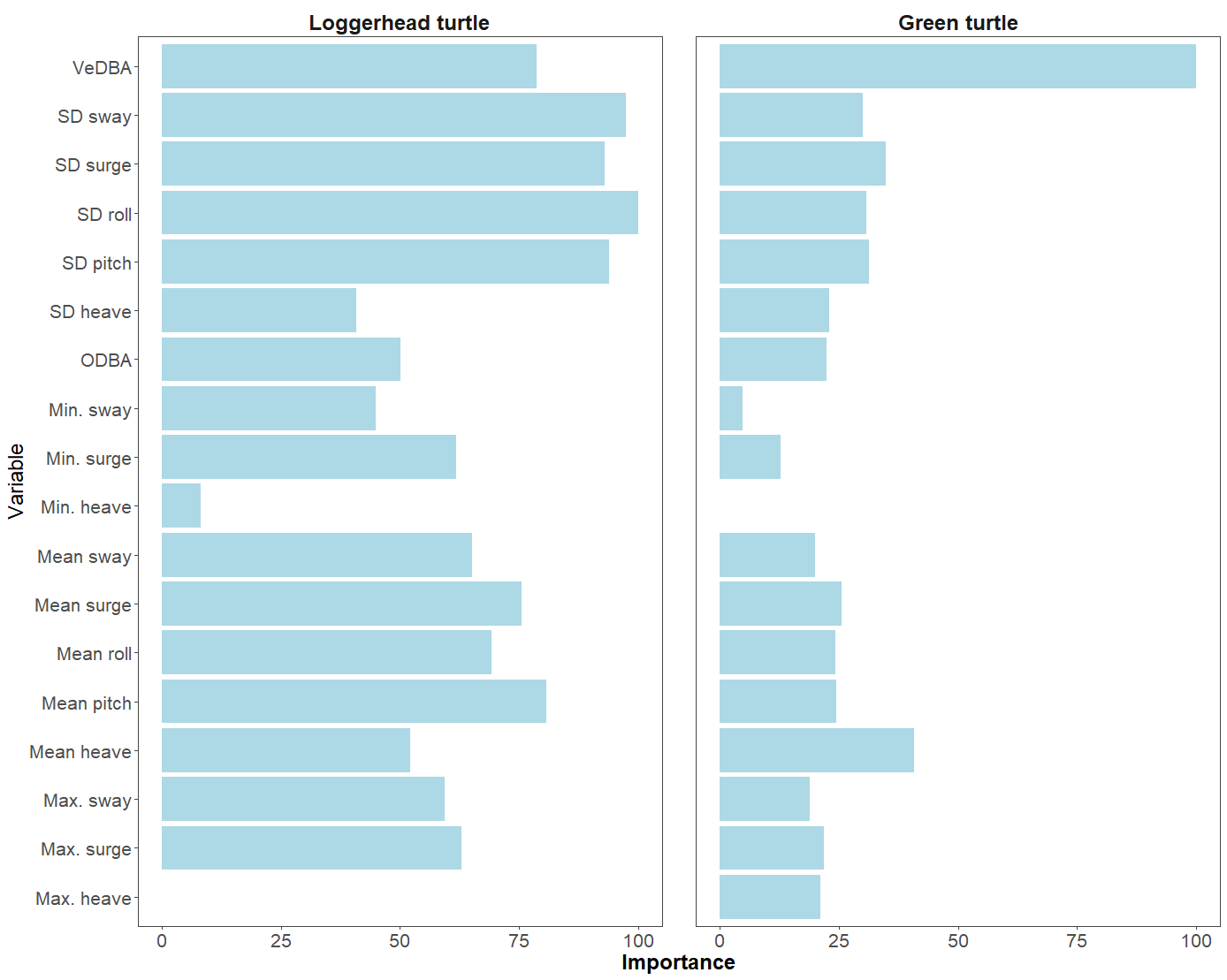


**Figure S3.** The variable importance of 18 summary metrics used to fit random forest classification models for 2 Hz sampling across 2 s window length for loggerhead turtles (left) and green turtles (right). Larger values indicate that metrics contributed more to the overall accuracy or predictive ability of the model. SD, standard deviation; Min., minimum; Max., maximum; ODBA, overall dynamic body acceleration; VeDBA, vectorial dynamic body acceleration


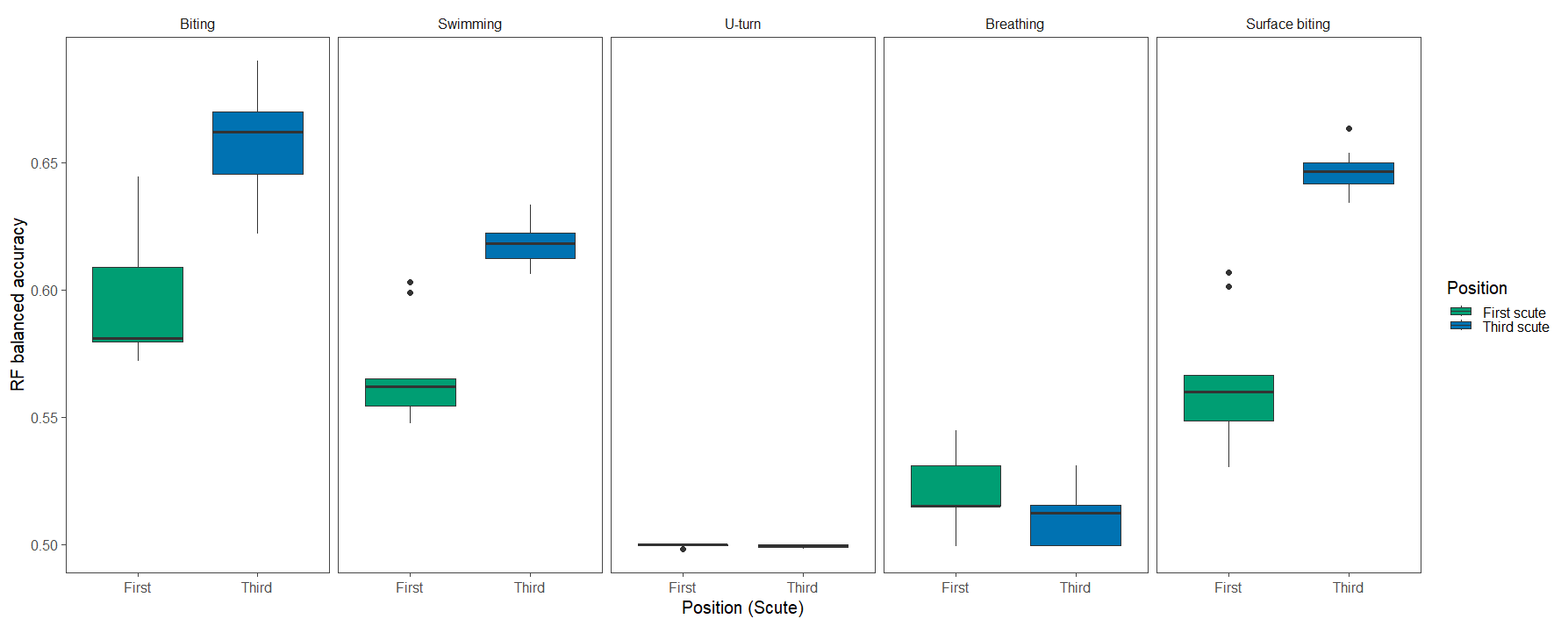

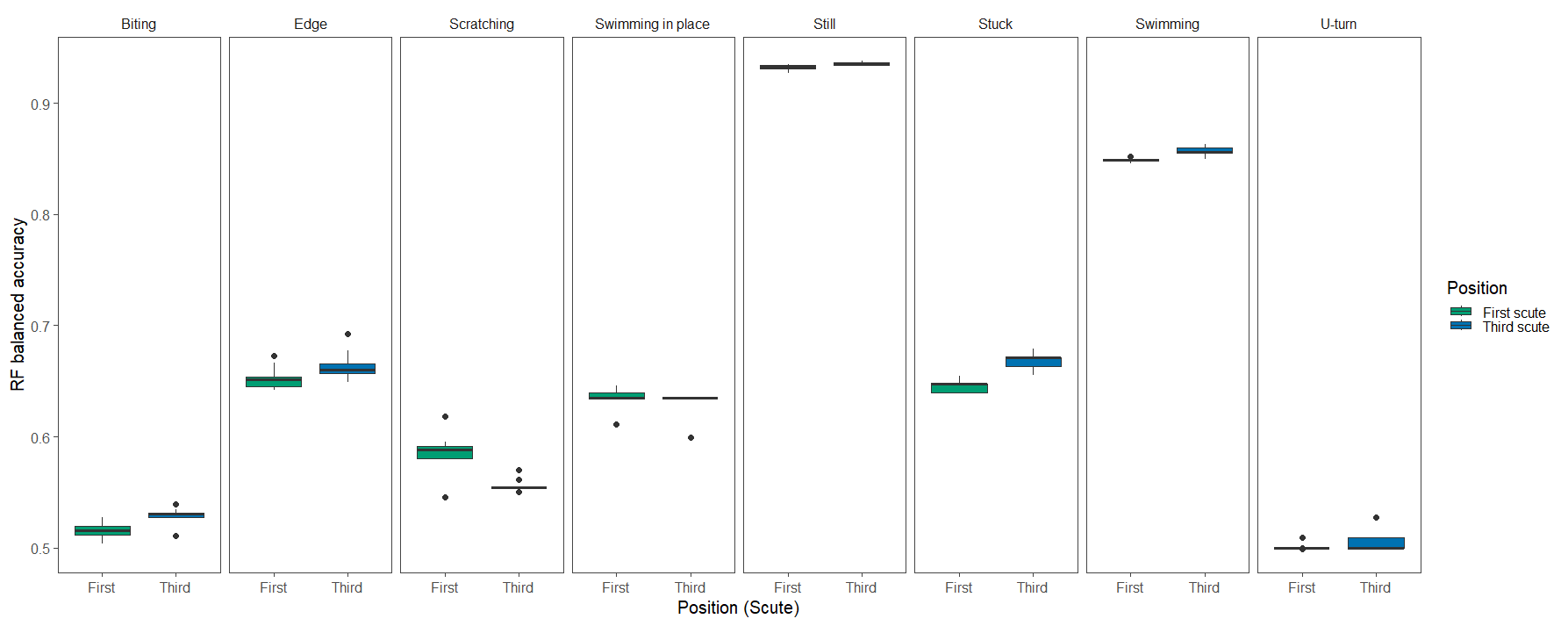


*******

*******

*******

*******

*******

**A.**

**B.**

**Figure S4.** Pairwise comparisons between balanced accuracy for position and behaviours for **(A)** loggerhead and **(B)** green turtles. Boxplots depict median relative accuracy levels and the 25th and 75th percentiles. Whiskers are 1.5 × the interquartile range. *** indicates a p-value  < 0.001, ** indicates a p-value  < 0.01.

| **Table S1.** Sea turtle biometrics and monitoring information. Filming occurred during daylight hours. Logger: Loggerhead sea turtle (*Caretta caretta*). Green: Green sea turtle (*Chelonia mydas*). CCL: curved carapace length. F: Female. M: Male. U: Unknown. Dur. Rec.: Duration of recording. Sta. GoPro: Static GoPro mounted above the tank. Pole GoPro: GoPro mounted on a telescopic pole. L. Leonardo: Little Leonardo animal-borne video camera. | | | | | | | |
| --- | --- | --- | --- | --- | --- | --- | --- |
| **Turtle ID** | **Species** | **CCL (cm)** | **Weight (Kg)** | **Sex** | **Dur. rec. (s)** | **Days** | **Method** |
| OG734 | Logger | 43 | 6.83 | F | 6941.67 | 1 | Sta. GoPro |
| OG738 | Logger | 39 | 7.04 | M | 7174.82 | 2 | Sta. GoPro |
| OG708 | Logger | 29 | 3.04 | F | 608.71 | 2 | Sta. GoPro |
| OG736 | Logger | 36.5 | 4.34 | M | 4872.89 | 2 | Sta. GoPro |
| OG739 | Logger | 40 | 8.21 | F | 4873.37 | 3 | Sta. GoPro |
| OG740 | Logger | 39 | 8.09 | M | 4552.58 | 2 | Sta. GoPro |
| OG744 | Logger | 36.5 | 6.15 | M | 11307.18 | 2 | Sta. GoPro |
| Amadeus | Green | 132 | 99 | F | 14821.16 | 2 | Pole GoPro/ L. Leonardo |
| Gijon | Green | 105,7 | 88 | M | 3497.08 | 2 | Pole GoPro/ L. Leonardo |
| Bubuja | Green | 48 | 13.45 | U | 304.73 | 1 | L. Leonardo |
| Nagoya | Green | 52,5 | 16.2 | U | 906.67 | 1 | L. Leonardo |
| Wasabi | Green | 48 | 13.45 | U | 1391.21 | 1 | Pole GoPro |
| Kawasaki | Green | 52,2 | 15 | U | 5522.49 | 1 | Pole GoPro |
| Tack | Green | 59 | 29 | U | 3200.96 | 1 | Pole GoPro |
| Enoshima | Green | 58 | 30 | U | 2957.5 | 1 | Pole GoPro |

**Table S2.** Minimum (*Min.*) and maximum (*Max.*) acceleration values obtained throughout deployments.

|  |  | **X axis (G)** | | **Y axis (G)** | | **Z axis (G)** | |
| --- | --- | --- | --- | --- | --- | --- | --- |
| **Species** | **Accelerometer Position** | *Min.* | *Max.* | *Min.* | *Max.* | *Min.* | *Max.* |
| Green turtles | *Scute 1* | -1.991 | 1.032 | -1.816 | 2.213 | -0.305 | 1.447 |
|  | *Scute 2* | -1.452 | 1 | -2.482 | 3.716 | -0.375 | 1.317 |
| Loggerhead turtles | *Scute 1* | -1.421 | 0.766 | -1.018 | 1.075 | -0.469 | 1.075 |
|  | *Scute 2* | -1.103 | 1.784 | -1.602 | 1.242 | 0.141 | 1.297 |

**Table S3.** Behaviours included in ethograms for loggerhead (L) and green (G) sea turtles. No. turtles: Number of turtles for which time was recoded after trimming. An asterisk (*) Indicates that some footage was obtained from Little Leonardo animal-borne video cameras. NA indicates behaviour was not of sufficient duration or was observed in too few individuals to include in RF models.

| **Behaviour** | **Description** | **Number of turtles** | **Time (s)** | **Window length (s)** |
| --- | --- | --- | --- | --- |
| *belly edge* | Swimming with belly on glass | L: NA  G: 1 | L: NA  G: 26.04 | L: NA  G: NA |
| *biting** | Biting object or food (eating) | L: 7  G: 6 | L: 888.44  G: 870.01 | L: 1 and 2  G: 1 and 2 |
| *bottom walking* | Turtle 'walks' along bottom with flippers | L: NA  G:3 | L: NA  G: 10.03 | L: NA  G: NA |
| *breathing** | Turtle breathing at surface | L: 7  G: 9 | L: 173.98  G: 290.43 | L: 1  G: 1 and 2 |
| *edge* | Turtle swims directly at the edge of tank | L: 7  G: 1 | L: 1,282.21  G: 1.01 | L: 1 and 2  G: NA |
| *flippers* | Using flippers to manipulate food | L: 4  G: NA | L: 79.38  G: NA | L: 1  G: NA |
| *gliding* | Swimming passively without flipper beat | L: 0  G: 5 | L: NA  G: 218.15 | L: 1  G: 1 |
| *interaction* | Interaction with another turtle | L: NA  G: 2 | L: NA  G: 49.06 | L: NA  G: NA |
| *grooming* | Turtle grooms self (flippers rub belly, back or face). | L: 3  G: 3 | L: 32.08  G: 48.15 | L: NA  G: NA |
| *locate food* | Turtle assumes direct vertical position with head pointed at floor | L: 3  G: 1 | L: 16.06  G: 14.03 | L: NA  G: NA |
| *scratching* | Turtle scratches self against object | L:6  G: 2 | L: 881.6  G: 2,250.35 | L: 1 and 2  G: NA |
| *still* | Flippers and body are static, includes assisted resting (resting anchored to an object) | L: 7  G: 2 | L: 16,105.45  G: 188.13 | L: 1 and 2  G: NA |
| *stuck* | Turtle stuck or caught on object, pulls trying to free self | L: 7  G: 1 | L: 453.45  G: 3.01 | L: 1 and 2  G: NA |
| *surface* | Carapace at surface, not breathing | L: 3  G: 3 | L: 13.1  G: 103.19 | L: NA  G: NA |
| *surface biting** | Biting water at surface | L: NA  G: 4 | L:  G: 656.54 | L: 1 and 2  G: 1 and 2 |
| *swimming* | Swimming | L: 7  G: 6 | L: 11,774.44  G: 8,990.75 | L: 1 and 2  G: 1 and 2 |
| *swimming in place* | Turtle is swimming but remains in same place | L: 7  G: NA | L: 603.55  G: NA | L: 1 and 2  G: NA |
| *u-turn* | Turtle turns sharply greater than 180 degrees | L: 7  G: 6 | L: 483.01  G: 217.84 | L: 1 and 2  G: 1 and 2 |

| **Table S4.** Summary metrics used in RF classification of accelerometer data. SD = Standard Deviation. Min. = minimum. Max. = maximum. Adapted from [2]. | |
| --- | --- |
| **Summary metric** | **Description** |
| Overall Dynamic Body Acceleration (ODBA, g) | Summation of absolute values of acceleration on each axis, after accounting for static g force. |
| Vectorial Dynamic Body Acceleration (VeDBA, g) | Vectorial sum of values of acceleration on each axis, after accounting for static g force. |
| Pitch (Mean and SD) | Rotational acceleration about the lateral axis. |
| Roll (Mean and SD) | Rotational acceleration about the longitudinal axis. |
| Surge (Mean, SD, Min., Max.) | Acceleration along the longitudinal axis (beak to tail) |
| Sway (Mean, SD, Min., Max.) | Acceleration along the lateral axis (flipper to flipper) |
| Heave (Mean, SD, Min., Max.) | Acceleration along the vertical axis (plastron to carapace) |

| **Table S5.** To assess the effect of sampling frequency (“Frequency”) on RF model balanced accuracy of individual behaviours, a beta regression model was conducted for loggerhead turtles. A window length of two seconds was used. Balanced accuracy (“*Balanced Accuracy*”) was included as the dependent variable. Sampling frequency (“*Frequency*”) and behaviour (“*Behaviour*”), were used as main effects with interactions. Overall model results for main effects and interaction terms are shown. Phi coefficients: Estimate = 2255.0, SE = 265.7, Z-value =8.486, P < 0.001. Pseudo R-squared: 0.997. SE= standard error. | | | | |
| --- | --- | --- | --- | --- |
|  | Estimate | SE | Z-value | p-value |
| (Intercept) | 0.090 | 0.013 | 7.091 | <.0001 |
| Edge | 0.536 | 0.018 | 29.082 | <.0001 |
| Scratching | 0.212 | 0.018 | 11.771 | <.0001 |
| Swimming in place | 0.483 | 0.018 | 26.382 | <.0001 |
| Still | 2.546 | 0.028 | 89.609 | <.0001 |
| Stuck | 0.562 | 0.018 | 30.517 | <.0001 |
| Swimming | 1.653 | 0.022 | 75.445 | <.0001 |
| U-turn | -0.093 | 0.018 | -5.204 | <.0001 |
| Frequency | -1 e -04 | 3 e -04 | -0.339 | 0.735 |
| Edge*Frequency | 0.001 | 5 e -04 | 2.969 | 0.003 |
| Scratching*Frequency | -0.001 | 5 e -04 | -1.318 | 0.188 |
| Swimming in place*Frequency | -0.001 | 5 e -04 | -2.408 | 0.016 |
| Still*Frequency | 5 e -04 | 0.001 | 0.615 | 0.539 |
| Stuck*Frequency | -1 e -04 | 5 e -04 | -0.309 | 0.758 |
| Swimming*Frequency | 0.001 | 0.001 | 1.162 | 0.245 |
| U-turn*Frequency | 0.001 | 5.E-04 | 1.576 | 0.115 |

| **Table S6.** To assess the effect of accelerometer position for individual behavioural accuracy a beta regression was conducted for loggerhead turtles. A window length of two seconds was used. As only position-specific differences within behaviours were of interest each combination of behaviour and scute position was treated as a unique level for the dependant variable (“Behaviour.position”). Balanced accuracy (“*Balanced Accuracy*”) was included as the dependent variable. Overall model results for main effects and interaction terms are shown. Phi coefficients: Estimate = 3271.4, SE = 385.5, Z-value =8.486, P < 0.001. Pseudo R-squared: 0.998. SE= standard error. | | | | |
| --- | --- | --- | --- | --- |
|  | Estimate | SE | Z-value | p-value |
| Edge.First scute | 0.060 | 0.012 | 5.146 | <.0001 |
| Scratching.First scute | 0.573 | 0.017 | 33.900 | <.0001 |
| Swimming in place.First scute | 0.286 | 0.017 | 17.230 | <.0001 |
| Still.First scute | 0.490 | 0.017 | 29.193 | <.0001 |
| Stuck.First scute | 2.560 | 0.026 | 98.768 | <.0001 |
| Swimming.First scute | 0.535 | 0.017 | 31.728 | <.0001 |
| U-turn.First scute | 1.664 | 0.020 | 83.178 | <.0001 |
| Biting.Third scute | -0.057 | 0.016 | -3.452 | <.0001 |
| Edge.Third scute | 0.055 | 0.017 | 3.319 | <.0001 |
| Scratching.Third scute | 0.622 | 0.017 | 36.648 | <.0001 |
| Swimming in place.Third scute | 0.164 | 0.017 | 9.911 | <.0001 |
| Still.Third scute | 0.475 | 0.017 | 28.321 | <.0001 |
| Stuck.Third scute | 2.612 | 0.026 | 98.974 | <.0001 |
| Swimming.Third scute | 0.638 | 0.017 | 37.528 | <.0001 |
| U-turn.Third scute | 1.730 | 0.020 | 85.171 | <.0001 |

| **Table S7.** Pair-wise comparison for accuracy of loggerhead turtle and accelerometer positioning. SE = Standard Error, DF= Degrees of freedom. ∞= infinite. | | | | | |
| --- | --- | --- | --- | --- | --- |
| Contrast | Estimate | SE | DF | z-ratio | p-value |
| Biting.First scute - Edge.First scute | -0.138 | 0.004 | ∞ | -34.366 | <.0001 |
| Biting.First scute - Scratching.First scute | -0.071 | 0.004 | ∞ | -17.289 | <.0001 |
| Biting.First scute - Swimming in place.First scute | -0.119 | 0.004 | ∞ | -29.487 | <.0001 |
| Biting.First scute - Still.First scute | -0.417 | 0.003 | ∞ | -127.963 | <.0001 |
| Biting.First scute - Stuck.First scute | -0.129 | 0.004 | ∞ | -32.108 | <.0001 |
| Biting.First scute - Swimming.First scute | -0.334 | 0.004 | ∞ | -93.101 | <.0001 |
| Biting.First scute - (U-turn.First scute) | 0.014 | 0.004 | ∞ | 3.452 | 0.046 |
| Biting.First scute - Biting.Third scute | -0.014 | 0.004 | ∞ | -3.320 | 0.070 |
| Biting.First scute - Edge.Third scute | -0.149 | 0.004 | ∞ | -37.242 | <.0001 |
| Biting.First scute - Scratching.Third scute | -0.041 | 0.004 | ∞ | -9.922 | <.0001 |
| Biting.First scute - Swimming in place.Third scute | -0.116 | 0.004 | ∞ | -28.589 | <.0001 |
| Biting.First scute - Still.Third scute | -0.420 | 0.003 | ∞ | -129.531 | <.0001 |
| Biting.First scute - Stuck.Third scute | -0.153 | 0.004 | ∞ | -38.167 | <.0001 |
| Biting.First scute - Swimming.Third scute | -0.342 | 0.004 | ∞ | -96.193 | <.0001 |
| Biting.First scute - (U-turn.Third scute) | 0.010 | 0.004 | ∞ | 2.422 | 0.530 |
| Edge.First scute - Scratching.First scute | 0.068 | 0.004 | ∞ | 16.914 | <.0001 |
| Edge.First scute - Swimming in place.First scute | 0.019 | 0.004 | ∞ | 4.802 | 0.000 |
| Edge.First scute - Still.First scute | -0.279 | 0.003 | ∞ | -88.935 | <.0001 |
| Edge.First scute - Stuck.First scute | 0.009 | 0.004 | ∞ | 2.219 | 0.684 |
| Edge.First scute - Swimming.First scute | -0.195 | 0.003 | ∞ | -56.293 | <.0001 |
| Edge.First scute - (U-turn.First scute) | 0.152 | 0.004 | ∞ | 37.894 | <.0001 |
| Edge.First scute - Biting.Third scute | 0.125 | 0.004 | ∞ | 30.988 | <.0001 |
| Edge.First scute - Edge.Third scute | -0.011 | 0.004 | ∞ | -2.819 | 0.258 |
| Edge.First scute - Scratching.Third scute | 0.097 | 0.004 | ∞ | 24.307 | <.0001 |
| Edge.First scute - Swimming in place.Third scute | 0.022 | 0.004 | ∞ | 5.688 | <.0001 |
| Edge.First scute - Still.Third scute | -0.282 | 0.003 | ∞ | -90.399 | <.0001 |
| Edge.First scute - Stuck.Third scute | -0.015 | 0.004 | ∞ | -3.724 | 0.018 |
| Edge.First scute - Swimming.Third scute | -0.204 | 0.003 | ∞ | -59.200 | <.0001 |
| Edge.First scute - (U-turn.Third scute) | 0.148 | 0.004 | ∞ | 36.840 | <.0001 |
| Scratching.First scute - Swimming in place.First scute | -0.049 | 0.004 | ∞ | -12.097 | <.0001 |
| Scratching.First scute - Still.First scute | -0.346 | 0.003 | ∞ | -107.506 | <.0001 |
| Scratching.First scute - Stuck.First scute | -0.059 | 0.004 | ∞ | -14.686 | <.0001 |
| Scratching.First scute - Swimming.First scute | -0.263 | 0.004 | ∞ | -74.074 | <.0001 |
| Scratching.First scute - (U-turn.First scute) | 0.085 | 0.004 | ∞ | 20.762 | <.0001 |
| Scratching.First scute - Biting.Third scute | 0.057 | 0.004 | ∞ | 13.956 | <.0001 |
| Scratching.First scute - Edge.Third scute | -0.079 | 0.004 | ∞ | -19.748 | <.0001 |
| Scratching.First scute - Scratching.Third scute | 0.030 | 0.004 | ∞ | 7.345 | <.0001 |
| Scratching.First scute - Swimming in place.Third scute | -0.045 | 0.004 | ∞ | -11.209 | <.0001 |
| Scratching.First scute - Still.Third scute | -0.350 | 0.003 | ∞ | -109.006 | <.0001 |
| Scratching.First scute - Stuck.Third scute | -0.082 | 0.004 | ∞ | -20.658 | <.0001 |
| Scratching.First scute - Swimming.Third scute | -0.271 | 0.004 | ∞ | -77.050 | <.0001 |
| Scratching.First scute - (U-turn.Third scute) | 0.081 | 0.004 | ∞ | 19.725 | <.0001 |
| Swimming in place.First scute - Still.First scute | -0.298 | 0.003 | ∞ | -94.095 | <.0001 |
| Swimming in place.First scute - Stuck.First scute | -0.010 | 0.004 | ∞ | -2.582 | 0.411 |
| Swimming in place.First scute - Swimming.First scute | -0.214 | 0.004 | ∞ | -61.282 | <.0001 |
| Swimming in place.First scute - (U-turn.First scute) | 0.133 | 0.004 | ∞ | 32.995 | <.0001 |
| Swimming in place.First scute - Biting.Third scute | 0.106 | 0.004 | ∞ | 26.124 | <.0001 |
| Swimming in place.First scute - Edge.Third scute | -0.030 | 0.004 | ∞ | -7.623 | <.0001 |
| Swimming in place.First scute - Scratching.Third scute | 0.079 | 0.004 | ∞ | 19.470 | <.0001 |
| Swimming in place.First scute - Swimming in place.Third scute | 0.004 | 0.004 | ∞ | 0.886 | 1.000 |
| Swimming in place.First scute - Still.Third scute | -0.301 | 0.003 | ∞ | -95.567 | <.0001 |
| Swimming in place.First scute - Stuck.Third scute | -0.033 | 0.004 | ∞ | -8.529 | <.0001 |
| Swimming in place.First scute - Swimming.Third scute | -0.223 | 0.003 | ∞ | -64.205 | <.0001 |
| Swimming in place.First scute - (U-turn.Third scute) | 0.129 | 0.004 | ∞ | 31.947 | <.0001 |
| Still.First scute - Stuck.First scute | 0.288 | 0.003 | ∞ | 91.310 | <.0001 |
| Still.First scute - Swimming.First scute | 0.084 | 0.003 | ∞ | 32.743 | <.0001 |
| Still.First scute - (U-turn.First scute) | 0.431 | 0.003 | ∞ | 132.278 | <.0001 |
| Still.First scute - Biting.Third scute | 0.403 | 0.003 | ∞ | 123.889 | <.0001 |
| Still.First scute - Edge.Third scute | 0.268 | 0.003 | ∞ | 85.943 | <.0001 |
| Still.First scute - Scratching.Third scute | 0.376 | 0.003 | ∞ | 115.999 | <.0001 |
| Still.First scute - Swimming in place.Third scute | 0.301 | 0.003 | ∞ | 95.057 | <.0001 |
| Still.First scute - Still.Third scute | -0.003 | 0.002 | ∞ | -1.573 | 0.973 |
| Still.First scute - Stuck.Third scute | 0.264 | 0.003 | ∞ | 84.989 | <.0001 |
| Still.First scute - Swimming.Third scute | 0.075 | 0.003 | ∞ | 29.924 | <.0001 |
| Still.First scute - (U-turn.Third scute) | 0.427 | 0.003 | ∞ | 130.982 | <.0001 |
| Stuck.First scute - Swimming.First scute | -0.204 | 0.003 | ∞ | -58.593 | <.0001 |
| Stuck.First scute - (U-turn.First scute) | 0.144 | 0.004 | ∞ | 35.626 | <.0001 |
| Stuck.First scute - Biting.Third scute | 0.116 | 0.004 | ∞ | 28.737 | <.0001 |
| Stuck.First scute - Edge.Third scute | -0.020 | 0.004 | ∞ | -5.039 | 0.000 |
| Stuck.First scute - Scratching.Third scute | 0.089 | 0.004 | ∞ | 22.070 | <.0001 |
| Stuck.First scute - Swimming in place.Third scute | 0.014 | 0.004 | ∞ | 3.468 | 0.044 |
| Stuck.First scute - Still.Third scute | -0.291 | 0.003 | ∞ | -92.777 | <.0001 |
| Stuck.First scute - Stuck.Third scute | -0.023 | 0.004 | ∞ | -5.945 | <.0001 |
| Stuck.First scute - Swimming.Third scute | -0.213 | 0.003 | ∞ | -61.507 | <.0001 |
| Stuck.First scute - (U-turn.Third scute) | 0.139 | 0.004 | ∞ | 34.575 | <.0001 |
| Swimming.First scute - (U-turn.First scute) | 0.348 | 0.004 | ∞ | 97.041 | <.0001 |
| Swimming.First scute - Biting.Third scute | 0.320 | 0.004 | ∞ | 89.359 | <.0001 |
| Swimming.First scute - Edge.Third scute | 0.184 | 0.003 | ∞ | 53.383 | <.0001 |
| Swimming.First scute - Scratching.Third scute | 0.293 | 0.004 | ∞ | 82.044 | <.0001 |
| Swimming.First scute - Swimming in place.Third scute | 0.218 | 0.004 | ∞ | 62.207 | <.0001 |
| Swimming.First scute - Still.Third scute | -0.087 | 0.003 | ∞ | -34.257 | <.0001 |
| Swimming.First scute - Stuck.Third scute | 0.181 | 0.003 | ∞ | 52.452 | <.0001 |
| Swimming.First scute - Swimming.Third scute | -0.008 | 0.003 | ∞ | -2.869 | 0.231 |
| Swimming.First scute - (U-turn.Third scute) | 0.344 | 0.004 | ∞ | 95.861 | <.0001 |
| (U-turn.First scute) - Biting.Third scute | -0.028 | 0.004 | ∞ | -6.773 | <.0001 |
| (U-turn.First scute) - Edge.Third scute | -0.163 | 0.004 | ∞ | -40.782 | <.0001 |
| (U-turn.First scute) - Scratching.Third scute | -0.055 | 0.004 | ∞ | -13.383 | <.0001 |
| (U-turn.First scute) - Swimming in place.Third scute | -0.130 | 0.004 | ∞ | -32.094 | <.0001 |
| (U-turn.First scute) - Still.Third scute | -0.435 | 0.003 | ∞ | -133.865 | <.0001 |
| (U-turn.First scute) - Stuck.Third scute | -0.167 | 0.004 | ∞ | -41.711 | <.0001 |
| (U-turn.First scute) - Swimming.Third scute | -0.356 | 0.004 | ∞ | -100.163 | <.0001 |
| (U-turn.First scute) - (U-turn.Third scute) | -0.004 | 0.004 | ∞ | -1.030 | 1.000 |
| Biting.Third scute - Edge.Third scute | -0.136 | 0.004 | ∞ | -33.853 | <.0001 |
| Biting.Third scute - Scratching.Third scute | -0.027 | 0.004 | ∞ | -6.599 | <.0001 |
| Biting.Third scute - Swimming in place.Third scute | -0.102 | 0.004 | ∞ | -25.229 | <.0001 |
| Biting.Third scute - Still.Third scute | -0.407 | 0.003 | ∞ | -125.441 | <.0001 |
| Biting.Third scute - Stuck.Third scute | -0.139 | 0.004 | ∞ | -34.775 | <.0001 |
| Biting.Third scute - Swimming.Third scute | -0.328 | 0.004 | ∞ | -92.424 | <.0001 |
| Biting.Third scute - (U-turn.Third scute) | 0.024 | 0.004 | ∞ | 5.743 | <.0001 |
| Edge.Third scute - Scratching.Third scute | 0.108 | 0.004 | ∞ | 27.155 | <.0001 |
| Edge.Third scute - Swimming in place.Third scute | 0.033 | 0.004 | ∞ | 8.510 | <.0001 |
| Edge.Third scute - Still.Third scute | -0.271 | 0.003 | ∞ | -87.405 | <.0001 |
| Edge.Third scute - Stuck.Third scute | -0.004 | 0.004 | ∞ | -0.905 | 1.000 |
| Edge.Third scute - Swimming.Third scute | -0.193 | 0.003 | ∞ | -56.283 | <.0001 |
| Edge.Third scute - (U-turn.Third scute) | 0.159 | 0.004 | ∞ | 39.724 | <.0001 |
| Scratching.Third scute - Swimming in place.Third scute | -0.075 | 0.004 | ∞ | -18.579 | <.0001 |
| Scratching.Third scute - Still.Third scute | -0.380 | 0.003 | ∞ | -117.522 | <.0001 |
| Scratching.Third scute - Stuck.Third scute | -0.112 | 0.004 | ∞ | -28.071 | <.0001 |
| Scratching.Third scute - Swimming.Third scute | -0.301 | 0.004 | ∞ | -85.062 | <.0001 |
| Scratching.Third scute - (U-turn.Third scute) | 0.051 | 0.004 | ∞ | 12.350 | <.0001 |
| Swimming in place.Third scute - Still.Third scute | -0.305 | 0.003 | ∞ | -96.530 | <.0001 |
| Swimming in place.Third scute - Stuck.Third scute | -0.037 | 0.004 | ∞ | -9.416 | <.0001 |
| Swimming in place.Third scute - Swimming.Third scute | -0.226 | 0.003 | ∞ | -65.134 | <.0001 |
| Swimming in place.Third scute - (U-turn.Third scute) | 0.126 | 0.004 | ∞ | 31.047 | <.0001 |
| Still.Third scute - Stuck.Third scute | 0.268 | 0.003 | ∞ | 86.449 | <.0001 |
| Still.Third scute - Swimming.Third scute | 0.078 | 0.002 | ∞ | 31.445 | <.0001 |
| Still.Third scute - (U-turn.Third scute) | 0.430 | 0.003 | ∞ | 132.563 | <.0001 |
| Stuck.Third scute - Swimming.Third scute | -0.189 | 0.003 | ∞ | -55.350 | <.0001 |
| Stuck.Third scute - (U-turn.Third scute) | 0.163 | 0.004 | ∞ | 40.652 | <.0001 |
| Swimming.Third scute - (U-turn.Third scute) | 0.352 | 0.004 | ∞ | 98.973 | <.0001 |

| **Table S8.** To assess the effect of sampling frequency (“Frequency”) on RF model balanced accuracy of individual behaviours, a beta regression model was conducted for green turtles. A window length of two seconds was used. Balanced accuracy (“*Balanced Accuracy*”) was included as the dependent variable. Sampling frequency (“*Frequency*”) and behaviour (“*Behaviour*”), were used as main effects with interactions. Overall model results for main effects and interaction terms are shown. Phi coefficients: Estimate = 256.20, SE = 38.12, Z-value =6.721, P < 0.001. Pseudo R-squared: 0.726. SE= standard error. | | | | |
| --- | --- | --- | --- | --- |
|  | Estimate | SE | Z-value | p-value |
| (Intercept) | 0.525 | 0.039 | 13.516 | <.0001 |
| Swimming | -0.157 | 0.054 | -2.885 | 0.004 |
| U-turn | -0.527 | 0.054 | -9.747 | <.0001 |
| Breathing | -0.468 | 0.054 | -8.661 | <.0001 |
| Surface biting | -0.117 | 0.055 | -2.134 | 0.033 |
| Frequency | -2 e -04 | 0.001 | -0.174 | 0.862 |
| Swimming*Frequency | 4 e -04 | 0.001 | 0.298 | 0.766 |
| U-turn*Frequency | 2 e -04 | 0.001 | 0.122 | 0.903 |
| Breathing*Frequency | 4 e -04 | 0.001 | 0.313 | 0.754 |
| Surface biting*Frequency | 0.001 | 0.001 | 0.659 | 0.510 |

| **Table S9.** To assess the effect of accelerometer position for individual behavioural accuracy a beta regression was conducted for green turtles. A window length of two seconds was used. As only position-specific differences within behaviours were of interest each combination of behaviour and scute position was treated as a unique level for the dependant variable (“Behaviour.position”). Balanced accuracy (“*Balanced Accuracy*”) was included as the dependent variable. Overall model results for main effects and interaction terms are shown. Phi coefficients: Estimate =1023.3, SE = 152.5, Z-value =6.711, P < 0.001. Pseudo R-squared: 0.932. SE= standard error. | | | | |
| --- | --- | --- | --- | --- |
|  | Estimate | SE | z-value | p-value |
| (Intercept) | 0.382 | 0.021 | 17.996 | <.0001 |
| Swimming.First scute | -0.114 | 0.030 | -3.827 | <.0001 |
| U-turn.First scute | -0.383 | 0.030 | -12.878 | <.0001 |
| Breathing.First scute | -0.294 | 0.030 | -9.894 | <.0001 |
| Surface biting.First scute | -0.128 | 0.030 | -4.282 | <.0001 |
| Biting.Third scute | 0.282 | 0.031 | 9.217 | <.0001 |
| Swimming.Third scute | 0.101 | 0.030 | 3.336 | 0.001 |
| U-turn.Third scute | -0.384 | 0.030 | -12.921 | <.0001 |
| Breathing.Third scute | -0.342 | 0.030 | -11.515 | <.0001 |
| Surface biting.Third scute | 0.221 | 0.030 | 7.254 | <.0001 |

| **Table S10.** Pair-wise comparison for accuracy of green turtle and accelerometer positioning. | | | | | |
| --- | --- | --- | --- | --- | --- |
| Contrast | Estimate | SE | DF | z-ratio | p-value |
| Biting.First scute - Swimming.First scute | 0.028 | 0.007 | ∞ | 3.829 | 0.005 |
| Biting.First scute - (U-turn.First scute) | 0.095 | 0.007 | ∞ | 12.957 | <.0001 |
| Biting.First scute - Breathing.First scute | 0.072 | 0.007 | ∞ | 9.930 | <.0001 |
| Biting.First scute - Surface biting.First scute | 0.031 | 0.007 | ∞ | 4.285 | 0.001 |
| Biting.First scute - Biting.Third scute | -0.066 | 0.007 | ∞ | -9.247 | <.0001 |
| Biting.First scute - Swimming.Third scute | -0.024 | 0.007 | ∞ | -3.338 | 0.029 |
| Biting.First scute - (U-turn.Third scute) | 0.095 | 0.007 | ∞ | 13.001 | <.0001 |
| Biting.First scute - Breathing.Third scute | 0.084 | 0.007 | ∞ | 11.571 | <.0001 |
| Biting.First scute - Surface biting.Third scute | -0.052 | 0.007 | ∞ | -7.269 | <.0001 |
| Swimming.First scute - (U-turn.First scute) | 0.067 | 0.007 | ∞ | 9.104 | <.0001 |
| Swimming.First scute - Breathing.First scute | 0.045 | 0.007 | ∞ | 6.089 | <.0001 |
| Swimming.First scute - Surface biting.First scute | 0.003 | 0.007 | ∞ | 0.456 | 1.000 |
| Swimming.First scute - Biting.Third scute | -0.094 | 0.007 | ∞ | -13.099 | <.0001 |
| Swimming.First scute - Swimming.Third scute | -0.052 | 0.007 | ∞ | -7.171 | <.0001 |
| Swimming.First scute - (U-turn.Third scute) | 0.067 | 0.007 | ∞ | 9.148 | <.0001 |
| Swimming.First scute - Breathing.Third scute | 0.057 | 0.007 | ∞ | 7.724 | <.0001 |
| Swimming.First scute - Surface biting.Third scute | -0.080 | 0.007 | ∞ | -11.114 | <.0001 |
| (U-turn.First scute) - Breathing.First scute | -0.022 | 0.007 | ∞ | -3.006 | 0.079 |
| (U-turn.First scute) - Surface biting.First scute | -0.063 | 0.007 | ∞ | -8.646 | <.0001 |
| (U-turn.First scute) - Biting.Third scute | -0.160 | 0.007 | ∞ | -22.343 | <.0001 |
| (U-turn.First scute) - Swimming.Third scute | -0.119 | 0.007 | ∞ | -16.332 | <.0001 |
| (U-turn.First scute) - (U-turn.Third scute) | 0.000 | 0.007 | ∞ | 0.043 | 1.000 |
| (U-turn.First scute) - Breathing.Third scute | -0.010 | 0.007 | ∞ | -1.375 | 0.935 |
| (U-turn.First scute) - Surface biting.Third scute | -0.146 | 0.007 | ∞ | -20.326 | <.0001 |
| Breathing.First scute - Surface biting.First scute | -0.041 | 0.007 | ∞ | -5.632 | <.0001 |
| Breathing.First scute - Biting.Third scute | -0.138 | 0.007 | ∞ | -19.268 | <.0001 |
| Breathing.First scute - Swimming.Third scute | -0.096 | 0.007 | ∞ | -13.291 | <.0001 |
| Breathing.First scute - (U-turn.Third scute) | 0.022 | 0.007 | ∞ | 3.050 | 0.070 |
| Breathing.First scute - Breathing.Third scute | 0.012 | 0.007 | ∞ | 1.631 | 0.833 |
| Breathing.First scute - Surface biting.Third scute | -0.124 | 0.007 | ∞ | -17.264 | <.0001 |
| Surface biting.First scute - Biting.Third scute | -0.097 | 0.007 | ∞ | -13.559 | <.0001 |
| Surface biting.First scute - Swimming.Third scute | -0.055 | 0.007 | ∞ | -7.628 | <.0001 |
| Surface biting.First scute - (U-turn.Third scute) | 0.064 | 0.007 | ∞ | 8.690 | <.0001 |
| Surface biting.First scute - Breathing.Third scute | 0.053 | 0.007 | ∞ | 7.267 | <.0001 |
| Surface biting.First scute - Surface biting.Third scute | -0.083 | 0.007 | ∞ | -11.573 | <.0001 |
| Biting.Third scute - Swimming.Third scute | 0.042 | 0.007 | ∞ | 5.900 | <.0001 |
| Biting.Third scute - (U-turn.Third scute) | 0.161 | 0.007 | ∞ | 22.388 | <.0001 |
| Biting.Third scute - Breathing.Third scute | 0.150 | 0.007 | ∞ | 20.934 | <.0001 |
| Biting.Third scute - Surface biting.Third scute | 0.014 | 0.007 | ∞ | 1.972 | 0.619 |
| Swimming.Third scute - (U-turn.Third scute) | 0.119 | 0.007 | ∞ | 16.376 | <.0001 |
| Swimming.Third scute - Breathing.Third scute | 0.108 | 0.007 | ∞ | 14.939 | <.0001 |
| Swimming.Third scute - Surface biting.Third scute | -0.028 | 0.007 | ∞ | -3.927 | 0.003 |
| (U-turn.Third scute) - Breathing.Third scute | -0.010 | 0.007 | ∞ | -1.418 | 0.922 |
| (U-turn.Third scute) - Surface biting.Third scute | -0.147 | 0.007 | ∞ | -20.371 | <.0001 |
| Breathing.Third scute - Surface biting.Third scute | -0.136 | 0.007 | ∞ | -18.923 | <.0001 |

Supplementary references

[1] Harvey-Carroll J, Crespo-Picazo J-L, Saubidet M, Robinson NJ, García-Párraga D, March D. Brushes and Shelters as Low-Cost Environmental Enrichment Devices for Loggerhead Turtles (Caretta caretta) During Rehabilitation. Chelonian Conserv Biol 2024;22. https://doi.org/10.2744/CCB-1596.1.

[2] Harvey-Carroll J, Carroll D, Trivella C-M, Connelly E. Classification of African ground pangolin based on accelerometer readouts: validation of bio-logging methods. Anim Biotelemetry 2024;12:22. https://doi.org/10.1186/s40317-024-00377-y.
